# Burden, and trends of breast cancer along with attributable risk factors in Gulf Cooperation Council countries from 1990 to 2019 and its projections

**DOI:** 10.1371/journal.pone.0331198

**Published:** 2025-10-06

**Authors:** Majed M. Ramadan, Heba A. Alkhatabi, Doaa Aboalola, Samkari Alaa, Rawiah A. Alsiary

**Affiliations:** 1 Population Health Research Section, King Abdullah International Medical Research Center, King Saud Bin Abdulaziz University for Health Sciences, Jeddah, Saudi Arabia; 2 Faculty of Applied Medical science, King Abdul Aziz University. Hematology Research Unit (HRU), King Fahad Medical Research Center (KFMRC); 3 Department of Cellular Therapy and Cancer Research, King Abdullah International Medical Research Center, Jeddah, Saudi Arabia; 4 King Saud Bin Abdulaziz University for Health Sciences, Jeddah, Saudi Arabia; 5 Department of Pathology and Laboratory Medicine, King Saud Bin Abdulaziz University for Health Science, King Abdulaziz Medical City, Ministry of National Guard- Health Affairs, Jeddah, Kingdom of Saudi Arabia; Fujian Provincial Hospital, CHINA

## Abstract

**Introduction:**

Breast cancer (BC) is a growing global public health concern, affecting millions of women worldwide. Gulf Cooperation Council (GCC) countries are no exception to this trend. Mortality rates in GCC nations are still high despite improvements in BC treatment. This article examines the changing picture of BC incidence, prevalence, and mortality in the GCC region from 1990 to 2019 and predictions up to 2030.

**Material and method:**

Using data from the Global Burden of Disease study, we analyzed BC incidence, prevalence, and mortality rates per 100,000 individuals across different age groups and countries.

**Results:**

The study reveals a significant rise in Age-Standardized Incidence Rates (ASIR) for breast cancer among females in Saudi Arabia from 1990 to 2019, with Oman experiencing the highest increase and Kuwait the highest decrease. Bahrain also saw a significant increase in male Age-standardized death rate (ASDR), despite all other countries experiencing a decrease. Also, the data demonstrated a statistically significant positive correlation between ASIR and Human Development Index (HDI), evident across all countries. Metabolic risk and tobacco use were identified as primary contributors. A ten-year BC prediction predicts a significant increase in female cases, with Saudi Arabia expected to experience the highest rise.

**Conclusion:**

This study underscores the urgent need for improved BC awareness, early detection through screening programs, enhanced access to quality healthcare services, and the addressing of sociocultural barriers in the GCC countries.

## Introduction

Breast cancer is one of the foremost public health concerns globally, affecting millions of women worldwide [1]. Gulf Cooperation Council (GCC) countries, comprising Saudi Arabia, Kuwait, the United Arab Emirates, Qatar, Bahrain, and Oman, are no exception to the rising prevalence of breast cancer [2]. The GCC countries have their unique demographic, economic, and healthcare contexts, which differ significantly from other Middle Eastern countries. The GCC countries have experienced rapid economic growth, urbanization, and lifestyle changes, leading to distinct epidemiological transitions and healthcare challenges, particularly in cancer burden. Despite considerable advancements in breast cancer management, mortality rates remain a significant concern in GCC countries. Research indicates that breast cancer incidence rates in GCC countries have been increasing steadily over the past few decades. This rise in incidence is associated with several factors, including changing lifestyle patterns, increased adoption of Westernized diets, evolving reproductive behaviors, urbanization, late-stage diagnosis, limited access to optimal treatment facilities, and socioeconomic disparities [2,3]. These factors may contribute to the observed trends, but the relationships are complex and influenced by a range of genetic, environmental, and healthcare system variables [4,5]. Projections suggest that breast cancer mortality rates in GCC countries will continue to rise if appropriate measures are not pursued to improve early detection, access to quality treatment, and patient outcomes; posing a significant public health challenge [6]. This knowledge can contribute to improved healthcare planning and resource allocation in the region.

In the GCC, breast cancer mortality, prevalence, and incidence are all on the rise, creating a rising public health concern. In addition to mortality, prevalence, and incidence, we included Disability-Adjusted Life Years (DALYs) to capture the overall burden of breast cancer by combining both fatal and non-fatal health outcomes. This metric provides a more comprehensive view of the disease’s impact on population health and supports prioritization of healthcare interventions in the GCC region. Therefore, in this paper, our main goal was to assess the estimated burden of breast cancer using the Global Burden of Disease (GBD) outline. The GBD provided comprehensive and easily accessible epidemiological data on 369 diseases and injuries, as well as 87 risk variables. This comprehensive data comprises 7 super-regions, 21 regions, and more than 200 countries and territories [7]. By incorporating the population projections and age-standardized breast cancer incidence data from 1990 to 2019 and applying forecasting techniques, we aimed to provide perceptions into the forthcoming new cases of breast cancer in GCC countries [8].

This study is among the first to conduct a thorough, country-specific analysis of breast cancer trends using Global Burden of Disease (GBD) data across multiple Gulf Cooperation Council (GCC) countries. While GBD data are globally accessible, few studies have provided this level of focused insight into the temporal and spatial variation of breast cancer burden in the Gulf region over a nearly three-decade period. Understanding the current trends and forecasting future trajectories of breast cancer incidence, prevalence, DALYs, and mortality in these countries is crucial for implementing effective prevention planning and treatment strategies. These efforts should focus on promoting awareness, early detection through screening programs, improving access to quality healthcare services, and addressing sociocultural barriers to optimal care. By addressing these factors, GCC countries can make significant progress in reducing the burden of breast cancer and improving patient outcomes.

## Methodology

### Data source, acquisition, and study design

In the current systemic analysis of the GBD study, the primary data was obtained from GBD 2019. The GBD provided inclusive and accessible epidemiological data on 369 diseases and injuries, as well as 87 risk factors, from 1990 to 2019. This comprehensive data encompasses 7 super-regions, 21 regions, and more than 200 countries and territories, and was obtained using a rigorous methodology previously described [9,10]. To obtain the necessary data, we utilized the Global Health Data Exchange (GHDx) tool (https://ghdx.healthdata.org/gbd-2019). The study used the following measures: breast cancer incidence, prevalence, and mortality for each selected country. The metrics for all selected measures (incidence, prevalence, Disability-adjusted life-years “DALYs”, and mortality) were the rate per 100,000 individuals. The study included the following risk factors that contributed to the burden of BC: dietary risks, low physical activity, metabolic risks, and tobacco. Although alcohol use is a known risk factor for breast cancer globally, in the GCC region, social, cultural, and legal factors result in significantly lower alcohol consumption compared to global averages [9–11]. Therefore, it was excluded from our analysis due to its low prevalence in this population. A detailed description of each risk factor’s definition, selection and the estimation of uncertainty interval are explained elsewhere (https://www.who.int/standards/classifications/classification-of-diseases) [11]. The human development index (HDI) data at the national level were collected from the United Nations Development Programmer [12]. HDI is a measure that evaluates a country’s progress in health, education, and standard of living. It offers a comprehensive view beyond economic growth, revealing a country’s overall quality of life and opportunities, providing a comprehensive understanding of a nation’s overall development.

### Study population

In this study, we included breast cancer population level data for Gulf Cooperation Council Countries (Saudi Arabia, Kuwait, the United Arab Emirates, Qatar, Bahrain, and Oman) from 1990 to 2019. The Global Burden of Disease study classified breast cancer according to the 10th revision of the International Classification of Diseases (ICD) 10 codes (i.e., C50-C50.9, D05-D05.9, D24-D24.9, D48.6, D49.3) [13]. This study includes both genders and utilized four intervals of age groups (20–24, 25–29, 30–34, 35–39, 40–44, 45–49, 50–54, 55–59, 60–64, 65–69, 70–74, 75–79, 80 + years old). The projections for the years 2020–2030 were obtained from the United Nations Department of Economic and Social Affairs/Population Division [14]. The population data was categorized by year, gender (both, female, male), and age (13 age groups spanning from 20 years old to 80 + years and older, in 5-year increments).

### Estimation of attributable burden

Estimating the attributable burden in GBD 2019 involves examining 560 pairs of risk-outcomes derived from systematically reviewed publications backed by credible or probable evidence. These 560 pairs are used for global estimates. The GBD 2019 utilized the Comparative Risk Assessment (CRA) method, extensively detailed in prior literature [9]. Within GBD 2019, four risk factors were identified as paired outcomes for breast cancer, including unhealthy diet (diet high in red meats), metabolic risk factors including both high body mass index (defined as BMI greater than 20–25 kg/m2), and elevated fasting plasma glucose (any level above 4.8–5.4 mmol/L), insufficient physical activity (< 3000–4500 metabolic equivalent (MET) minutes/week), and tobacco use (including smoking, and exposure to secondhand smoke). Data on the proportion of breast cancer deaths and DALYs attributable to these risk factors for six countries were extracted from GBD 2019.

### Data analysis

The incidence of BC by country was quantified using the estimated annual percentage change (EAPC) and its corresponding 95% uncertainty interval, as reported in the previous literature [15]. This approach allowed for a comprehensive assessment of the trends and variability in BC incidence over time and across different geographical locations. The Age-standardized rate (ASR) for breast cancer, already reported in the GBD data, were utilized in our analysis. We selected two-time points, specifically 1990 and 2019 to calculate the age-standardized incidence percentage change of BC for each selected risk factor, and country. The formula for calculating EAPC is as follows:


$$ASR=\;\frac{\sum_{i=\text{0}}^{A}aiwi}{\sum_{i=\text{1}}^{A}wi}\times \text{1}\text{0}\text{0},\text{0}\text{0}\text{0}$$

(ai, denotes the ith age class, and the number of persons (or weight) (wi) in the same age subgroup i of the selected reference standard population).

The trends in age-standardized rates serve as crucial indicators of evolving disease patterns and risk factors. To capture these trends over time, we employed the widely recognized ASR-EAPC measurement [16,17]. EAPC, developed to characterize ASR trends over defined time frames, operates under the assumption of a linear relationship between ASR and the natural logarithm.

In the equation Y = α + βX + ε, Y represents the natural logarithm of the age-standardized rate “(ln (ASR)”, X represents the calendar year, and ∊ represents the error term. In this formula, the coefficient β determines whether ASR trends are positive or negative. The formula for calculating the EAPC is as follows:


EAPC=100×[exp(β)−1].

The linear model offers 95% confidence intervals (CIs) [18,19]. When the EAPC and the lower CI are positive, the ASR tends to increase. Conversely, in a downward trend [16], ASR shows a negative EAPC and an upper CI. Projected numbers and ASRs of incidence until 2030 were extrapolated using the Bayesian age-period-cohort (BAPC) model [20,21]. Among several statistical methods evaluated, including the smooth spline model, and Poisson regression, the BAPC model emerged as the most suitable for projecting the cancer burden, particularly for short-term projections. Bayesian Poisson spatial model was employed to guarantee balanced estimations and precise results for every province. By using data from neighboring areas this spatial modeling technique makes it possible to produce accurate estimates for places with little or no data. All analyses were performed using SAS statistical software version 9.4 (SAS Institute Inc. Cary, NC).

## Results

### Age standardized annual percentage change of breast cancer incidence, death, and DALY’s in GCC countries from 1990 to 2019

Saudi Arabia had a significant increase in BC Age-Standardized Incidence Rates (ASIR) per 100,000 for both sexes (64.39%) and females (65.49%) from 1990 to 2019, accompanied by notable rises in Age-standardized death rate (ASDR) (22.95%) and DALY’s (24.12%) for both sexes, as well as for female ASDR (25.59%), and DALY’s (24.6%). Oman had the highest increase in ASDR per 100,000 for females (31.17%), while Kuwait reported the highest decrease in ASDR for females (−36.28%) per 100,000 from 1990–2019. All countries experienced a decrease in male ASDR except for Bahrain where a 39.13% increase of male ASDR from 1990 to 2019 (Table 1).

**Table 1:** Age standardized annual percentage change of incidence, death, and DALY’s burden rates in GCC countries from 1990 to 2019.

	ASIR^1,2^			ASDR ^1,2^			DALYs ^1,2^		
	1990 (95%UI)3	2019 (95%UI)	Change %	1990 (95%UI)	2019 (95%UI)	Change, %	1990 (95%UI)	2019 (95%UI)	Change %
Saudi Arabia									
Both	6.11 (4.47,8.25)	17.16 (12.79,22.69)	64.39	4.5 (3.27,6.22)	5.84 (4.47, 7.6)	22.95	134.91 (97.22,185.03)	177.79 (132.71,177.79)	24.12
Female	14.86 (10.75,20.12)	43.06 (31.94,57.1)	65.49	10.67 (7.72,14.83)	14.34 (10.91,18.71)	25.59	336.4 (241.47,461.45)	446.14 (332.23,589.45	24.6
Male	0.32 (0.21,0.46)	0.38 (0.25,0.54)	15.78	0.27 (0.18,0.39)	0.21 (0.13,0.29)	−28.57	6.28 (4.17,9.08)	4.43 (2.98,6.24)	−41/76
Kuwait									
Both	15.52 (17.06,14.09)	18.11 (14.6,23.15)	14.3	7.06 (6.41,7.71)	5.46 (4.45,6.91)	−29.30	194.58 (178.11,212.91)	152.51 (124.01,195.61)	−27.59
Female	41.34 (37.56,45.76)	42.76 (34.38,54.65)	3.32	17.73 (16.13,19.39)	13.01 (10.55,16.58)	−36.28	526.52 (481.84,578.84)	358.84 (290.26,461.81)	−46.73
Male	0.5 (0.38,0.65)	0.51 (0.35,0.75)	1.96	0.32 (0.25,0.41)	0.24 (0.17,0.35)	−33.33	6.78 (5.43,8.55)	5.00 (3.59,7.24)	−36.6
Qatar									
Both	16.79 (12.55,22.51)	25.45 (19.8,32.1)	34.02	10.83 (7.94,14.71)	8.98 (7.08,11.17)	−20.60	258.12 (195.57,341.36)	209.71 (162.47,262.54)	−23.08
Female	48.92 (36.98,64.61)	103.72 (80.21,131.21)	52.83	28.21 (20.87,37.95)	36.21 (28.89,45.77)	22.09	797.43 (605.15,1045.64)	856.37 (662.49.107.64)	6.88
Male	0.76 (0.46,1.15)	0.86 (0.52,1028)	11.62	0.54 (0.33,0.81)	0.42 (0.27,0.62)	−28.57	13.00 (7.96,19.27)	8.35 (5.16,12.02)	−55.69
Bahrain									
Both	19.82 (16.86,23.22)	26.15 (21.07,31.89)	24.21	12.48 (10.72,14.44)	10.61 (8.68,12.28)	−17.62	332.99 (283.43,390.33)	253.44 (203.02,311.01)	−31.39
Female	46.76 (38.89,53.96)	67.49 (54.04,83.05)	30.71	27.38 (23.51,31.81)	25.16 (20.44,30.45	−8.82	788.57 (670.99,925.05)	668.34 (533.29,827.71)	−17.99
Male	0.19 (0.13,0.26)	0.45 (0.28,0.66)	57.77	0.14 (0.1,0.2)	0.23 (0.14,0.34)	39.13	3.38 (2.44,4.58)	5.05 (3.2,7.33)	33.07
Oman									
Both	9.31 (6.62,13.32)	19.47 (16.31,22.93)	52.18	5.76 (4.11,8.21)	7.65 (6.47,9.02)	24.71	153.82 (109.11,220.59)	187.98 (157.76.222.13)	18.17
Female	19.28 (13.15,28.41)	44.65 (36.83,52.87)	56.82	10.93 (7.52,16.16)	15.88 (13.29,18.91)	31.17	326.03 (221.56,479.83)	434.82 (359.14,519.01)	25.02
Male	2.31 (1.46,3.37)	2.54 (1.67,3.67)	9.06	1.84 (1.17,2.66)	1.42 (0.96,2.07)	−29.58	36.91 (23.65,52.78)	26.27 (17.71,37.04)	−40.5
United Arab Emirates									
Both	13.98 (10.04,19.62)	15.03 (11.5,19.41)	6.99	9.38 (6.76,13.28)	7.27 (5.61,9.31)	−29.02	245.35 (176.62,341.31)	204.37 (156.21,266.21)	−20.05
Female	40.77 (28.7,57.79)	57.47 (43.29,73.69)	29.06	25.49 (17.94,36.31)	26.19 (20.03,33.55)	2.67	743.75 (526.28,1041.73)	790.97 (593.96,1020.86)	5.97
Male	1.21 (0.52,2.07)	1.12 (0.52,2.04)	−8.04	0.96 (0.41,1.65)	0.7 (0.33,1,24)	−37.14	21.86 (9.83,36.67)	17.11 (8.07,30.67)	−27.76

^1^per 100,000.

^2^ASDR: Age-standardized death rate, ASIR: Age-standardized incidence rate, *DALYs:* Disability-adjusted life-years, BC: breast cancer.

^3^UI: Uncertainty interval.

### Sex and age distribution in prevalence, incidence, death, and DALY’s rate in 2019

**Fig 1:**
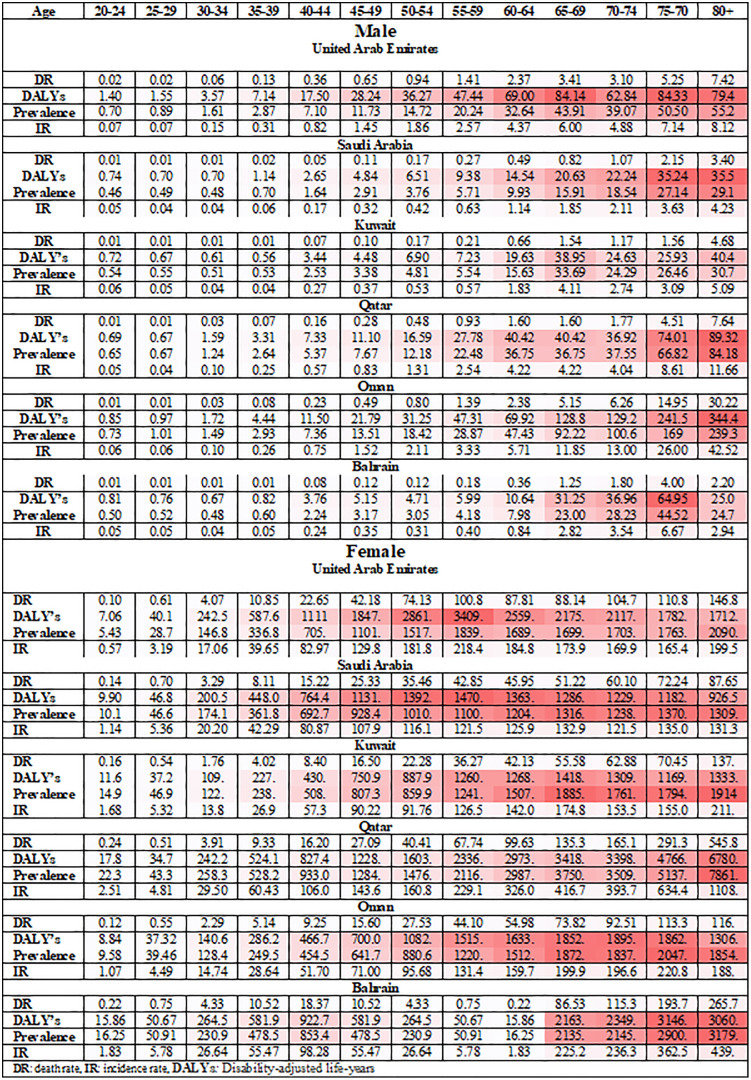
Decomposition of age for prevalence, IR, DR, and DALY’s rate in 2019 by sex (Heatmap).

Significant disparities in the age and sex distribution of BC were observed in the counts of prevalence, incidence, death, and DALY’s and the corresponding rate. In 2019, the IR, DR, and DALY’s for males across all countries were higher in older age groups starting from 60 + compared to age groups starting from 35 + ages in females (Fig 1). The highest IR, and DR and DALY’s for males were in Oman IR (42.52 per 100,000), and DR (30.22 per 100,000), and DALY’s (344.4 per 100,000), and prevalence (239.3 per 100,000). In 2019, United Arab Emirates had the highest female ASIR (1839. per 100,000) DR (100.8 per 100,000), and DALY’s (3409 per 100,000), and prevalence (218.4 per 100,000) in the age group 55–59 (Fig 1).

### Trends in ASIR, ASDR, prevalence and DALY’s by sex from 1990 to 2019

**Fig 2:**
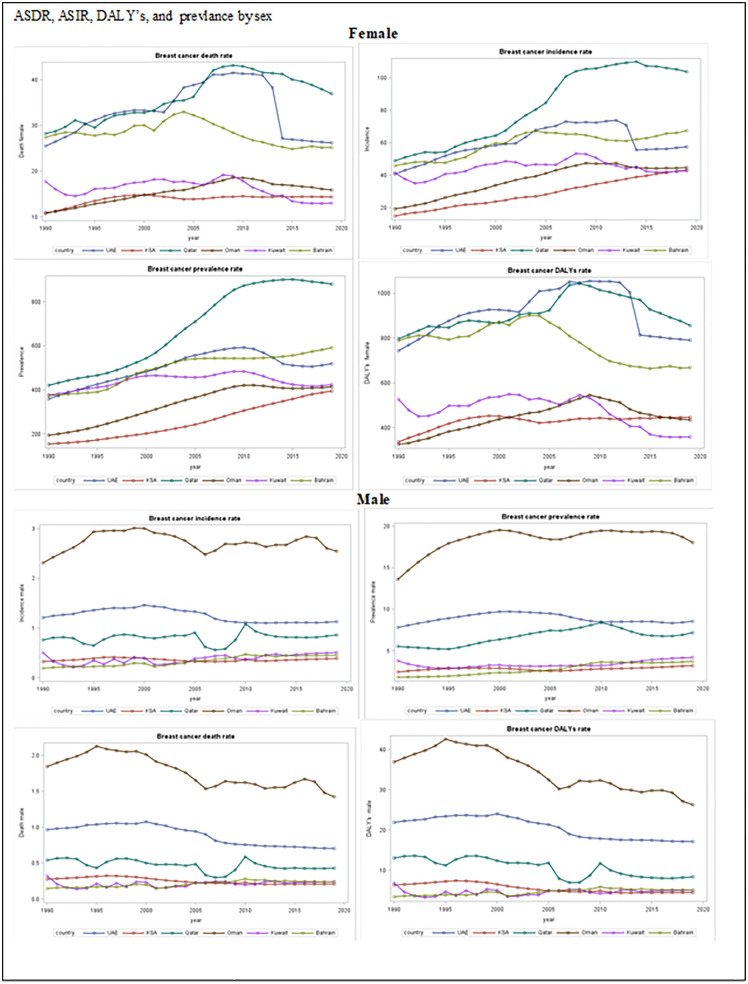
Temporal trends for age standardized DALY’s, ASIR, ASDR and prevalence by sex (1990 to 2019).

Between 1990 and 2019, the ASIR, ASDR, prevalence, and DALYs of female and male BC increased greatly in all GCC countries. Among these countries, Saudi Arabia had the highest uptrend increase in ASIR among female (65.49%) and Bahrain had the highest ASIR in male (57.77%). In 2019, Qatar exhibited the highest ASIR (103.72 per 100,000; 95%CI 80.21,131.21). The highest uptrend increases in ASDR, and DALY’s in female was in Oman (31.17%, 25.02% respectively), followed by Saudi Arabia (25.59%), while the highest ASDR uptrend increase was in Bahrain (39.13%). In 2019, the highest female ASDR was in Qatar (36.21 per 100,000; 95%UI 28.89,45.77) (Table 1, Fig 2). Oman exhibited the highest prevalence uptrend increases in male (9.41%), and Qatar exhibited the highest prevalence female uptrend increases (52.83%) over the study period (Table 1, S1 Table, Fig 2).

### Association of ASIR, ASDR, prevalence, and age standardized DALYs rate with HDI

**Fig 3:**
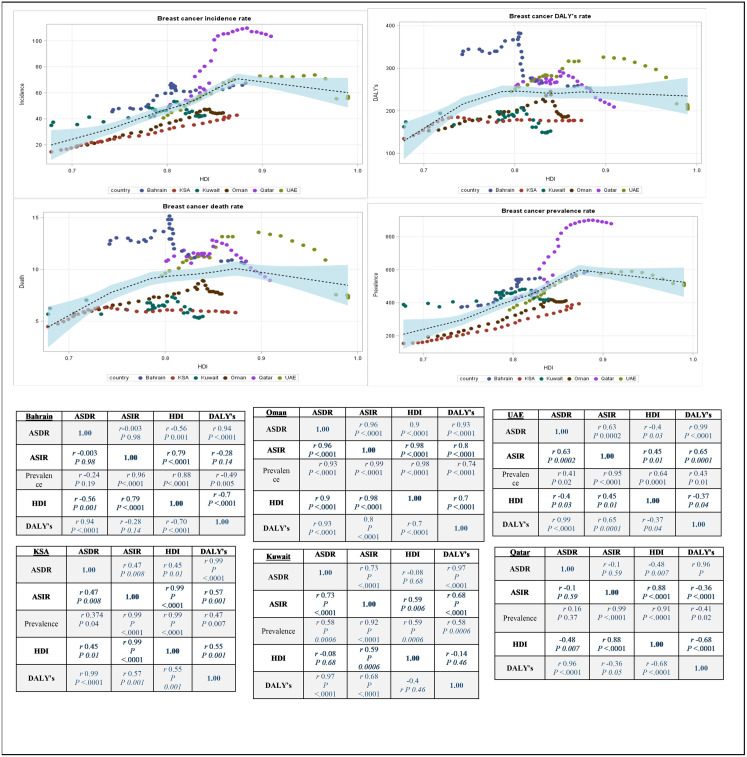
Spearman correlation between ASIR, ASDR, DALY’s, and prevalence with HDI.

There is a statistically significant positive correlation between ASIR and HDI across all countries with the highest correlation was in Saudi Arabia (r = 0.99; *p* < .0001). While a fluctuation was observed in the correlations between ASDR, DALY’s and HDI across all countries. The highest significant negative correlation between ASDR and HDI was in Bahrain (r = −0.56; *p* *<* 0.001). The highest significant negative correlation between DALY’s and HDI was in Qatar (r = −0.68; *p* < .0001) (Fig 3).

### BC ASDR attributable to risk factors

**Fig 4:**
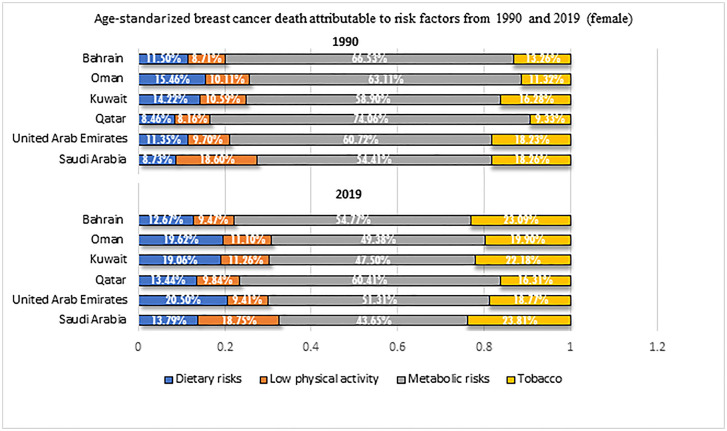
Age-standardized percentage changes in breast cancer death attributable to risk factors from 1990 and 2019 (female).

During 1990–2019, metabolic risk followed by tobacco consumption were the largest contributor of BC ASDR for females for all included countries. Metabolic risk contribution of BC ASDR was the highest in Qatar with (74.06%) in 1990, and (60.41%) in 2019. In 2019, tobacco contribution of BC ASDR for females was the highest in Kuwait (22.16%). Between 1990–2019, all GCC countries experienced an increase in metabolic risk contribution. The highest increase of metabolic risk was in Oman with (27.79%; 95% 27.1–28.5). Deaths associated with low physical activity; dietary risk displayed different trends across the included countries (Table 2, Fig 4).

**Table 2:** Age standardized percentage changes in breast cancer death attributable to risk factors from 1990 and 2019 (female)^5.^

Risk factors	Saudi Arabia (95% UI)^1^	United Arab Emirates (95% UI)	Qatar (95% UI)	Kuwait (95% UI)	Oman (95% UI)	Bahrain (95% UI)
1990						
Dietary risks^2^	0.13 (0.00002,0.27)	0.13 (−0.00007, 0.28)	0.13 (−0.00004, 0.28)	0.13 (−0.00006 −0.28)	0.13 (−0.00004,0.28)	0.13 (−0.00005 −0.28)
Low physical Activity	0.03 (0.0005, 0.005)	0.028 (0.005, 0.05)	0.036 (0.007, 0.064)	0.044 (0.008, 0.07)	0.031 (0.006, 0.056)	0.03 (0.006, 0.058)
Metabolic risks^3^	0.08 (−0.01,0.17)	0.11 (−0.01,0.25)	0.13 (−0.02,0.26)	0.13 (−0.017, 0.27)	0.088 (−0.011,0.18)	0.12 (−0.017, 0.25)
Tobacco	0.014 (0.0005, 0.027)	0.02 (0.001, 0.04)	0.016 (−0.0001,0.03)	0.027 (0.0023, 0.053)	0.017 (0.0025,0.032)	0.024 (0.006, 0.042)
2019						
Dietary risks	0.12 (−0.00006,0.27)	0.13 (−0.00003, 0.28)	0.13 (−0.00004, 0.28	0.13 (−0.00006 −0.28)	0.13 (−0.00004,0.28)	0.13 (−0.00005 - 0.28)
Low physical activity	0.03 (0.0006, 0.005)	0.032 (0.006, 0.061)	0.036 (0.007, 0.064)	0.044 (0.008, 0.07)	0.031 (0.006, 0.056)	0.033 (0.006, 0.059)
Metabolic risks	0.14 (−0.02, 0.28)	0.2 (−0.0310.39)	0.2 (−0.03,0.38)	0.18 (−0.025, 0.34)	0.15 (−0.02, 0.31)	0.17 (−0.029, 0.34)
Tobacco^4^	0.016 (0.0002, 0.03)	0.015 (0.0007,0.03)	0.016 (−0.0001,0.03	0.022 (0.0007, 0.044)	0.014 (0.0017, 0.027)	0.021 (0.0035, 0.037)
Changes in percentage from 1990 to 2019						
Dietary risks	−7.69 (**−**7.710 – −7.67)	----^6^	----	----	----	----
Low physical activity	----	14.28 (14.26–14.3)	----	----	----	----
Metabolic risks	75 (74.98–75.02)	81.81 (81.79–81.83)	53.84 (53.82–53.86)	14.28 (14.26–14.3)	87.5 (87.48–87.52)	41.66 (41.64–41.68)
Tobacco	14.28 (14.26–14.3)	−25 (**−**25.02– −24.98)	----	−22.72 (**−**22.74 – −22.7)	−21.42 (**−**21.44 – −21.4)	−14.28 (**−**14.3 – −14.26)

^1^Uncertainty interval (UI).

^2^Dietary risks refer to diet high in red meat.

^3^Metabolic risk factors include high fasting plasma glucose (FPG) and high body mass index (BMI).

^4^Tobacco include smoking and secondhand smoke.

^5^Dietary risks, and Metabolic risk are not available for male.

^6^no percentage change.

### Predictions of BC incidence in CGG countries by sex

**Fig 5:**
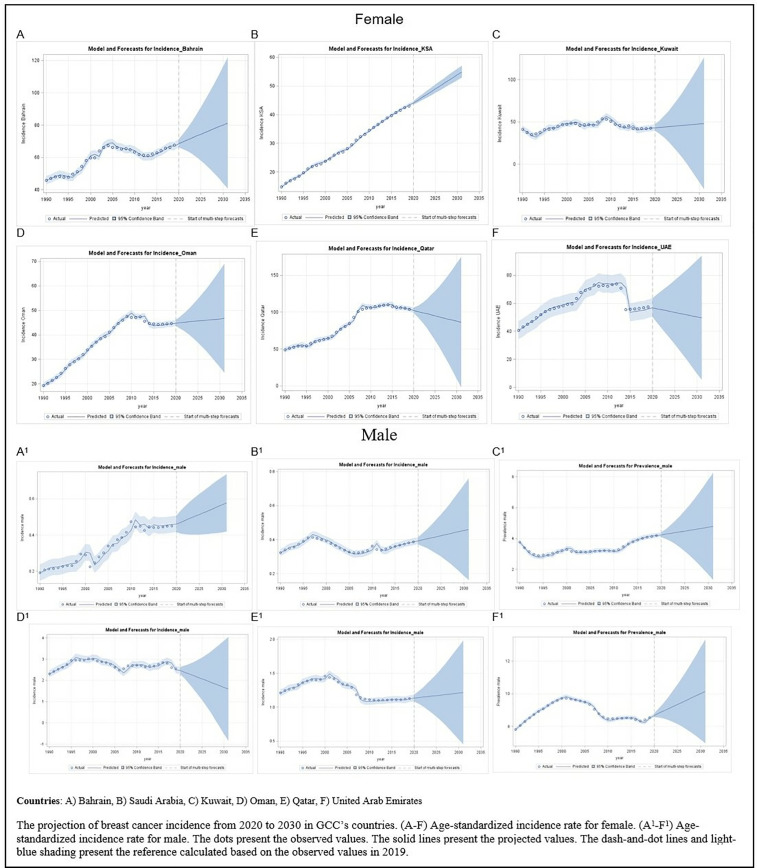
ASIR prediction from 2020 to 2030 by sex.

We compared incidence rate calculated by BAPC model with observed rate from the GBD 2019 between 1990 and 2019 and found that the highest BC projected new cases among females was in Saudi Arabia with (nearly 20%, 9441 new cases) increase in the ten years projection. Whereas Bahrain showed the highest projected BC new cases among males with approximately 21% increase (Table 3, Fig 5).

**Table 3:** Age standardized projection of new cases of breast cancer in GCC countries from 2020 to 2030^3.^

Year	United Arab Emirates			Saudi Arabia			Kuwait			Oman			Bahrain			Qatar		
	Value1	95%CI2		value	95%CI		value	95%CI		value	95%CI		value	95%CI		value	95%CI	
Female																		
2020	56.82	50.29	63.35	44.06	43.46	44.66	43.20	39.36	47.05	44.83	43.58	46.08	68.64	66.06	71.22	102.26	98.78	105.73
2021	56.17	46.17	66.16	45.05	44.20	45.89	43.64	35.83	51.45	45.00	42.58	47.41	69.79	65.00	74.57	100.79	93.03	108.56
2022	55.51	42.31	68.71	46.04	45.00	47.07	44.08	31.59	56.58	45.17	41.40	48.93	70.93	63.65	78.22	99.33	86.34	112.32
2023	54.85	38.50	71.21	47.03	45.83	48.23	44.52	26.7	62.34	45.34	40.05	50.62	72.08	62.01	82.15	97.87	78.86	116.89
2024	54.20	34.65	73.75	48.02	46.68	49.36	44.96	21.23	68.69	45.51	38.55	52.46	73.22	60.10	86.35	96.41	70.67	122.15
2025	53.54	30.73	76.35	49.01	47.54	50.48	45.4	15.23	75.57	45.67	36.90	54.45	74.37	57.95	90.79	94.95	61.84	128.06
2026	52.89	26.73	79.04	50.00	48.42	51.59	45.84	8.75	82.93	45.84	35.12	56.57	75.52	55.57	95.47	93.49	52.43	134.56
2027	52.23	22.63	81.83	50.99	49.30	52.69	46.28	1.81	90.75	46.01	33.21	58.81	76.66	52.96	100.36	92.03	42.46	141.6
2028	51.57	18.44	84.71	51.98	50.18	53.78	46.72	−5.56	99.00	46.18	31.19	61.18	77.81	50.15	105.46	90.57	31.98	149.16
2029	50.92	14.15	87.69	52.97	51.08	54.87	47.15	−13.35	107.66	46.35	29.05	63.65	78.96	47.15	110.76	89.11	21.	157.2
2030	50.26	9.76	90.77	53.96	51.97	55.96	47.59	−21.52	116.71	46.52	26.81	66.23	80.1	43.96	116.25	87.65	9.59	165.71
Male																		
2020	1.14	1.08	1.19	0.39	0.37	0.41	0.49	0.36	0.62	2.47	2.30	2.63	0.46	0.42	0.51	0.85	0.64	1.05
2021	1.14	1.05	1.24	0.40	0.36	0.44	0.50	0.37	0.63	2.39	2.08	2.69	0.47	0.41	0.54	0.85	0.64	1.06
2022	1.15	1.01	1.29	0.41	0.35	0.46	0.51	0.38	0.64	2.31	1.85	2.77	0.48	0.40	0.56	0.85	0.65	1.06
2023	1.16	0.96	1.35	0.41	0.33	0.49	0.51	0.38	0.64	2.23	1.60	2.86	0.49	0.40	0.58	0.85	0.65	1.06
2024	1.16	0.91	1.41	0.42	0.32	0.52	0.52	0.39	0.65	2.15	1.34	2.96	0.50	0.40	0.61	0.86	0.65	1.06
2025	1.17	0.86	1.48	0.42	0.30	0.55	0.53	0.40	0.66	2.07	1.06	3.08	0.51	0.40	0.63	0.86	0.65	1.07
2026	1.18	0.80	1.56	0.43	0.28	0.58	0.53	0.40	0.66	1.99	0.77	3.21	0.52	0.40	0.65	0.86	0.66	1.07
2027	1.19	0.74	1.64	0.44	0.26	0.61	0.54	0.41	0.67	1.91	0.47	3.36	0.54	0.41	0.66	0.86	0.66	1.07
2028	1.19	0.67	1.72	0.44	0.24	0.65	0.55	0.42	0.68	1.83	0.16	3.51	0.55	0.41	0.68	0.87	0.66	1.07
2029	1.20	0.60	1.80	0.45	0.21	0.68	0.55	0.43	0.68	1.76	−0.17	3.68	0.56	0.41	0.7	0.87	0.66	1.08
2030	1.21	0.53	1.89	0.45	0.19	0.72	0.56	0.43	0.69	1.68	−0.51	3.86	0.57	0.41	0.72	0.87	0.67	1.08

^1^precited value.

^2^95% confidence interval.

^3^values per 100,000.

## Discussion

The current investigation highlights gender-specific trends in mortality and incidence, such as substantial increase in ASIR for breast cancer among females in Saudi Arabia from 1990 to 2019. Regarding ASDR per 100,000 in females, Oman experienced the highest increase, while Kuwait experienced the highest decrease. Bahrain saw a significant increase in male ASDR between 1990 and 2019, in contrast to all other countries where male ASDR decreased over this period. The study also establishes a positive correlation between the Human Development Index (HDI) and ASIR, highlighting the impact of socioeconomic development on breast cancer burden. Metabolic risk and tobacco use were identified as the primary contributors to ASDR of breast cancer in all studied countries, enhancing understanding of modifiable risk factors and adding policy relevance by pointing to preventable drivers of disease burden in the region. The ten-year BC prediction indicates a significant increase in female cases, with Saudi Arabia expected to experience the highest rise. Understanding current trends and anticipating future breast cancer incidence, prevalence, and mortality in these countries is critical for developing effective prevention and treatment plans. The novelty lies in the integration of longitudinal epidemiological data, regional specificity, risk factor attribution, and predictive modeling, delivering a powerful tool for policymakers, researchers, and clinicians in the Gulf.

### ASIR trends in women

The results of this study show a significant increase in ASIR for breast cancer among females and both sex in Saudi Arabia between 1990 and 2019. This finding reflects the collaborative efforts of the Saudi government and healthcare organizations to offer easily accessible screening services and advocate for regular mammograms to facilitate early detection [22,23]. Also, Saudi Arabia established a National Plan Cancer Control to ensure early detection and reduction of cancer-related mortality rates. This initiative employs a variety of strategies, including improving access to diagnostic and treatment services, improving healthcare infrastructure, and conducting public awareness campaigns. The National Plan for Cancer Control demonstrates the government’s commitment to reducing the cancer burden and improving patient outcomes across the country [24].

Another finding is that Oman experienced the highest increase in ASDR per 100,000 for females (31.17%), while Kuwait experienced the highest decrease (−36.28%) per 100,000 from 1990–2019. These significant variations in ASDR among females reflect the challenges and success in cancer control efforts in the GCC region. Further research is required to assess the various factors influencing healthcare systems and public health initiatives in Oman, Kuwait, and other GCC countries, such as healthcare infrastructure, access to healthcare services, advances in diagnostic capabilities, and changes in lifestyle factors or environmental exposures that contribute to higher mortality rates. This variation in ASDR between Kuwait and Oman could be attributed to the fact that Kuwait has a better health system capacity than Oman as explained by The World Health Organization (WHO). WHO’s report on Kuwait’s cancer patient care reveals high-quality patient care, with a population-based cancer registry (PBCR) of 11.2 available per 10,000 cancer patients. The report also shows a high availability of external beam radiotherapy units (photon and electron) and diagnostic imaging capabilities, with 273.6 CT and 234.5 MRI scanners per 10,000 cancer patients, and 30.7 PET or PET/CT scanners per 10,000 cancer patients [25]. In contrast, there was significantly less medical equipment in Oman that was needed for cancer detection and treatment. Oman had 45.2 mammographs, 138.5 CT scanners, 111.4 MRI scanners, and 6 PET or PET/CT scanners per 10,000 cancer patients [26]. While specific comparative data on medical resource distribution are limited, the availability and accessibility of comprehensive cancer treatment, including specialized oncology services and advanced medical technologies, are crucial for improving patient outcomes. Differences in these resources between Oman and Kuwait may influence ASDR trends, possibly due to variations in healthcare infrastructure and resource allocation. Therefore, this disparity suggests that Kuwaiti patients may have better access to essential radiotherapy services, potentially leading to improved cancer outcomes. Furthermore, Kuwait has established organized screening programs aimed at early cancer detection. The Kuwait National Mammography Screening Program (KNMSP), initiated in 2014, offers nationwide breast cancer screening for women aged 40 and above. Over five years, the program screened 14,773 women, which facilitated early treatment and likely contributed to the observed decrease in ASDR among Kuwaiti women [27].

### ASDR trends in men

Bahrain experienced a high increase in male ASDR (39.13%), whereas all other GCC countries reported declines in male ASDR between 1990 and 2019. The available research on male breast cancer is quite limited. A single study of occult triple-negative male breast cancer was reported from Bahrain [28]. This finding was unexpected and necessitated more inquiry to determine why Bahrain has a different pattern in male ASDR compared to the other GCC states. The finding could be attributed to differences in awareness, healthcare infrastructure, screening differences, public attitudes, and data reporting processes between nations. Another explanation for this finding is that Bahrain’s healthcare expenditures are higher than the GCC regional average. In addition, unlike neighboring countries, Bahraini physicians and nurses constitute the majority of the country’s healthcare personnel [27]. Moreover, the UN World Population Prospects data show that Bahrain’s population structure is shifting with increasing proportions of older adults. Therefore, although ASDR adjusts for age, Bahrain’s relatively aging male population and longer life expectancy may contribute to a higher ASCDR [29]. In addition, Bahrain exhibits high rates of obesity, diabetes, and hypertension, which are significant risk factors for various cancers. The rising prevalence of these conditions may also contribute to increased cancer mortality [29].

Our Data demonstrated that male breast cancer occurrences among people over the age of 60 in studied counties have increased significantly in recent decades, however, research in this field remains restricted. A substantial correlation between negative emotions and heightened risk of breast cancer initiation was uncovered in a recent study, with indications that these emotions could also impact the prognosis of the disease [30]. The rising frequency of depression among the elderly may be connected to an increase in male BC occurrences [31]. Despite its low incidence rate, our findings highlight the urgent need for increased emphasis on male BC in the GCC area. Age- and sex-specific DALY patterns reveal critical disparities: male DALYs are concentrated in older age groups (60+), with Oman and the UAE showing the highest respective rates, while in females the elevated IR, DR, and DALYs emerges from the age group of 35 years and above. This could be due to improved awarekness in the younger generation with the availability of social media, marketing for BC campaigns, and increased literacy compared to the older female generation. In UAE, most of the BC cases occurred in women under the age of 50. In a recent study, death rate dropped after the age of 65, this could be explained by the low and young population of the UAE as well as illiteracy in older patients [29].

### Association between ASIR and HDI

The statistically substantial positive connection between ASIR and HDI, which is observed in all countries, points to an intriguing relationship between socioeconomic development and the incidence of breast cancer. This result suggests that as countries progress in terms of human development, which can be measured by factors like income, education, and access to healthcare, the incidence rates of not only breast cancer but also other diseases like kidney cancer [32] and chronic respiratory disorders [33] tend to grow. This association likely reflects complex interactions among lifestyle changes, reproductive behaviors, and healthcare access that accompany socioeconomic progress. For example, higher HDI is often associated with delayed childbearing, fewer pregnancies, and increased use of hormonal therapies—all factors correlated with breast cancer risk [4,5]. Moreover, diets in higher-HDI settings tend to include more processed and high-fat foods, which have been associated with increased breast cancer risk in observational studies [34]. Urbanization and sedentary lifestyles, which are linked to better socioeconomic position and common in more developed regions, contribute to metabolic changes such as obesity and insulin resistance, which are linked to breast cancer through hormonal and inflammatory [35–37]. Furthermore, higher-HDI countries may have better screening systems and healthcare infrastructure, which might result in increased reported incidence by enabling earlier and more frequent detection [38]. This phenomenon is reflected globally, with high-income countries reporting breast cancer incidence rates exceeding 80 per 100,000, while low-income countries report rates below 40 per 100,000 [39]. BC is a major public health problem, especially in low-income countries that have limited capacity to provide breast cancer programs comparable to high-income countries [40,41]. While ASIR rises with HDI, we also observed fluctuations in correlations between ASDR, DALYs, and HDI across countries highlight the complex interplay of factors such as healthcare infrastructure, public health initiatives, socioeconomic inequalities, and cultural influences [42–44]. These findings emphasize the necessity of incorporating socioeconomic indicators like HDI when designing breast cancer prevention, screening, and treatment strategies. Tailored interventions that address country-specific health system strengths and gaps are essential to reduce disparities in breast cancer outcomes both within and between GCC countries.

### Metabolic and lifestyle factors in breast cancer: evidence and insights

Our data show that metabolic risk including elevated body mass index and high fasting plasma glucose—was the main attributable of ASDR of breast cancer in the GCC. These metabolic conditions may promote carcinogenesis via hormonal dysregulation, chronic inflammation, and altered adipokine profiles [45–47]. This finding is consistent with Smolarz’s research (2022), which highlights the increase in breast cancer incidence in developed countries as a result of lifestyle variables such poor eating habits, sedentary lifestyles, and stress [41]. Additionally, our results suggest that the second attributed factor of ASDR of breast cancer among females in all studied countries from 1990 to 2019 was tobacco use including both active smoking and secondhand smoke exposure. These associations align with findings from recent research identifying smoking as a major cause of breast cancer in North African and Middle Eastern nations [48]. Tobacco use, correlates with increased breast cancer risk and worse outcomes, potentially through DNA damage and immune modulation [49,50]. Other well-established risk factors—such as reproductive history (early menarche, late menopause, parity), breastfeeding practices, and family history of breast cancer—also contribute to individual risk but were not fully captured in this population-level analysis [41,51]. A healthy diet, abstaining from smoking, controlling body weight, and participating in regular exercise can reduce the number of risk factors linked to breast cancer. To successfully address these issues, initiatives supporting healthy lifestyles are essential, ranging from early education to government-sponsored advertising efforts.

### Projections for the next decade

Our analysis of the ten-year BC prediction shows a disturbing trend: the number of new cases among females is likely to increase during the next decade. Among the studied countries, Saudi Arabia is expected to experience the greatest rise, with nearly 20% increase in incidents rate comparing to than in the previous decade, reaching an estimated 9,441 additional cases. This finding was consistent with the worldwide potential rise. It was predicted that by 2040, both the incidence and death rates of breast cancer will have increased dramatically. The estimate predicts a whopping 40% growth in new cases each year, totaling almost 3 million cases worldwide. Furthermore, breast cancer-related mortality is predicted to increase by 50%, resulting in about 1 million deaths each year [39]. Demographic changes such as an aging population and changes in reproductive methods may have an influence on the reported patterns. As populations age and women postpone or opt to have fewer children, the risk of breast cancer increases. It was recommended that increasing females’ awareness about breast cancer and how to avoid risk factors, along with early detection of breast cancer, can decrease the death rate from this disease [39,52,53]. The predicted increase differed between GCC countries for both females and men, with Saudi Arabia predicting the highest number of new BC cases among females and Bahrain among males. It is critical to extensively investigate these disparities to understand the different BC risk factors across the studied countries, such as lifestyle, healthcare systems, and government protection strategies [54,55]. Saudi Arabia and Bahrain must prioritize tackling this major health risk.

### Policy implications and recommendations

By including DALYs alongside incidence and mortality, this study sheds light on the full impact of breast cancer beyond just the number of cases and deaths. Our results show that DALY trends closely follow mortality patterns, underscoring the critical need for early detection and better cancer care. These findings provide valuable guidance for policymakers to prioritize resources and develop health programs that not only aim to improve survival but also enhance quality of life. To tackle these challenges, several key actions are needed. First and foremost, public awareness campaigns stressing breast cancer risk factors and early detection should be combined with the implementation of comprehensive screening programs, which should specifically target high-risk populations. Secondly, in order to provide prompt access to diagnostic and treatment services, investments in healthcare infrastructure are essential. Thirdly, in addition to developing and implementing national policies that support efforts to prevent breast cancer, specific interventions for male breast cancer patients should also be created. To maximize effect, cooperation between government organizations, and healthcare professionals is also crucial. Finally, in order to lower obstacles to screening and treatment and increase access to care, socioeconomic inequities must be addressed.

### Strength and limitations

Utilizing the data from GBD 2019, this research offers a comprehensive evaluation of breast cancer burden and future projection. Notably, it represents the first attempt, to the best of our knowledge, to forecast the trajectory of age-standardized breast cancer incidence trends in GCC countries up to 2030. These findings provide valuable insight for policymakers, as they can inform future cancer control planning, resource allocation, and targeted prevention strategies. The identification of tobacco use and metabolic risks as leading contributors to breast cancer burden enables the development of region-specific health policies aimed at modifiable risk factors. Despite its strength, this study also bears certain limitations. Firstly, the predictive models did not account for specific significant risk factors such as family history in the estimation of future breast cancer incidence. Thus, the study is subject to confounding bias. Secondly, GBD data as a population-level data can lead to ecological fallacy, where associations observed at the population level are incorrectly attributed to individuals within that population. Thirdly, it’s important to acknowledge that our study shares the general limitations associated with GBD research, including factors like the availability and quality of primary data.

Furthermore, estimates from the Global Burden of Disease (GBD) study rely on statistical models that help fill gaps in data, especially in countries where health information systems are still developing. In the Gulf Cooperation Council (GCC) countries, cancer registry systems differ in terms of coverage, completeness, and data quality. Challenges such as underreporting, inconsistent data collection methods, and delays in reporting can affect the accuracy of local cancer data. These issues may influence the reliability of GBD estimates, possibly leading to under- or overestimation of cancer burden in the region. Therefore, it is important to interpret these estimates with caution. Strengthening cancer registration systems and improving data collection practices across GCC countries will be essential for producing more accurate and useful public health information in the future.

## Supporting information

S1 Table Temporal trends of ASIR, ASDR, Prevalence, and DALY’s by sex. (DOCX)

## Abbreviations

DR: Death rate
IR: Incidence rate
ASDR: Age-standardized death rate
ASIR: Age-standardized incidence rate
ASR: Age-standardized rate
BAPC: Bayesian age-period-cohort
BAMP: Bayesian age-period-cohort modeling and prediction
BC: Breast cancer
BMI: Body mass index
CI: Confidence interval
DALYs: Disability-adjusted life-years
EAPC: Estimated annual percentage change
FPG: Fasting plasma glucose
GBD: Global Burden of Disease
HDI: human development index
UI: Uncertainty interval

## References

[1] GBD 2019 Cancer Risk Factors Collaborators. The global burden of cancer attributable to risk factors, 2010-19: a systematic analysis for the global burden of disease study 2019. Lancet. 2022;400(10352):563–91. doi: 10.1016/S0140-6736(22)01438-6 35988567 PMC9395583

[2] Tanner LTA, Cheung KL. Correlation between breast cancer and lifestyle within the Gulf Cooperation Council countries: A systematic review. World J Clin Oncol. 2020;11(4):217–42. doi: 10.5306/wjco.v11.i4.217 32355643 PMC7186238

[3] Wang F, Luo L, McLafferty S. Healthcare access, socioeconomic factors and late-stage cancer diagnosis: an exploratory spatial analysis and public policy implication. Int J Public Pol. 2010;5(2–3):237–58. 23316251 10.1504/IJPP.2010.030606 PMC3540777

[4] Collaborative Group on Hormonal Factors in Breast Cancer. Menarche, menopause, and breast cancer risk: individual participant meta-analysis, including 118 964 women with breast cancer from 117 epidemiological studies. Lancet Oncol. 2012;13(11):1141–51. doi: 10.1016/S1470-2045(12)70425-4 23084519 PMC3488186

[5] Clavel-Chapelon F, E3N-EPIC Group. Differential effects of reproductive factors on the risk of pre- and postmenopausal breast cancer. Results from a large cohort of French women. Br J Cancer. 2002;86(5):723–7. doi: 10.1038/sj.bjc.6600124 11875733 PMC2230628

[6] Albeshan SM, Mackey MG, Hossain SZ, Alfuraih AA, Brennan PC. Breast Cancer Epidemiology in Gulf Cooperation Council Countries: A Regional and International Comparison. Clin Breast Cancer. 2018;18(3):e381–92. doi: 10.1016/j.clbc.2017.07.006 28781021

[7] Five insights from the Global Burden of Disease Study 2019. Lancet. 2020;396(10258):1135–59. 33069324 10.1016/S0140-6736(20)31404-5 PMC7116361

[8] Mohammed EM. High Number of Familial Breast Cancer Cases in the Arabian Gulf Countries: Investigating the Reasons. Breast Cancer (Auckl). 2022;16:11782234221107121. doi: 10.1177/11782234221107121 35783595 PMC9243472

[9] GBD 2019 Risk Factors Collaborators. Global burden of 87 risk factors in 204 countries and territories, 1990-2019: a systematic analysis for the Global Burden of Disease Study 2019. Lancet. 2020;396(10258):1223–49. doi: 10.1016/S0140-6736(20)30752-2 33069327 PMC7566194

[10] Global burden of 369 diseases and injuries in 204 countries and territories, 1990-2019: a systematic analysis for the global burden of disease study 2019. Lancet. 2020;396(10258):1204–22. 33069326 10.1016/S0140-6736(20)30925-9 PMC7567026

[11] Network GB o DC. Global Burden of Disease Study 2019 (GBD 2019) Results. Seattle, United States: Institute for Health Metrics and Evaluation (IHME). 2020. http://ghdx.healthdata.org/gbd-results-tool

[12] Undp. Human development report 2021-22.

[13] World Health O. ICD-10: international statistical classification of diseases and related health problems: tenth revision. Geneva: World Health Organization. 2004.

[14] United Nations D o E a SA. Population Prospects 2019, Online Edition. Revision 1. 2019. https://population.un.org/wpp/Download/Standard/Population

[15] Liu Z, Jiang Y, Yuan H, Fang Q, Cai N, Suo C, et al. The trends in incidence of primary liver cancer caused by specific etiologies: results from the global burden of disease study 2016 and implications for liver cancer prevention. J Hepatol. 2019;70(4):674–83. doi: 10.1016/j.jhep.2018.12.001 30543829

[16] Li N, et al. Global burden of breast cancer and attributable risk factors in 195 countries and territories, from 1990 to 2017: results from the global burden of disease study 2017. J Hematol Oncol. 2019;12(1):1–12. 31864424 10.1186/s13045-019-0828-0 PMC6925497

[17] Liu Z, Jiang Y, Yuan H, Fang Q, Cai N, Suo C, et al. The trends in incidence of primary liver cancer caused by specific etiologies: Results from the Global Burden of Disease Study 2016 and implications for liver cancer prevention. J Hepatol. 2019;70(4):674–83. doi: 10.1016/j.jhep.2018.12.001 30543829

[18] Gao S, Yang W-S, Bray F, Va P, Zhang W, Gao J, et al. Declining rates of hepatocellular carcinoma in urban Shanghai: incidence trends in 1976-2005. Eur J Epidemiol. 2012;27(1):39–46. doi: 10.1007/s10654-011-9636-8 22160277 PMC5477645

[19] Hankey BF, Ries LA, Kosary CL, Feuer EJ, Merrill RM, Clegg LX, et al. Partitioning linear trends in age-adjusted rates. Cancer Causes Control. 2000;11(1):31–5. doi: 10.1023/a:1008953201688 10680727

[20] Rahimzadeh S, Burczynska B, Ahmadvand A, Sheidaei A, Khademioureh S, Pazhuheian F, et al. Geographical and socioeconomic inequalities in female breast cancer incidence and mortality in Iran: A Bayesian spatial analysis of registry data. PLoS One. 2021;16(3):e0248723. doi: 10.1371/journal.pone.0248723 33730079 PMC7968648

[21] Vollset SE, Goren E, Yuan C-W, Cao J, Smith AE, Hsiao T, et al. Fertility, mortality, migration, and population scenarios for 195 countries and territories from 2017 to 2100: a forecasting analysis for the Global Burden of Disease Study. Lancet. 2020;396(10258):1285–306. doi: 10.1016/S0140-6736(20)30677-2 32679112 PMC7561721

[22] Omalkhair Abualkhair AS, Al Harthy B, Tahan F, Baroum I. Clinical Practice Guideline on the Use of Screening Strategies for the Detection of Breast Cancer. 2014. https://www.moh.gov.sa/en/Ministry/Structure/Programs/TCP/Documents/8.%20Breast%20Cancer%20-%20Use%20of%20Screening%20Strategies%20for%20the%20Detection%20of%20Breast%20Cancer.pdf

[23] Abulkhair OA, Al Tahan FM, Young SE, Musaad SM, Jazieh A-RM. The first national public breast cancer screening program in Saudi Arabia. Ann Saudi Med. 2010;30(5):350–7. doi: 10.4103/0256-4947.67078 20697170 PMC2941246

[24] Health MO. National Plan Cancer Control. 2014. https://www.iccp-portal.org/system/files/plans/SAU_B5_National%20Plan%20Cancer%20Control.pdf

[25] Organization WH. Cancer Kuwait 2020 Country Profile. 2020. https://www.who.int/publications/m/item/cancer-kwt-2020

[26] Organization WH. Cancer Oman 2020 country profile. 2020. https://www.who.int/publications/m/item/cancer-omn-2020

[27] Kingdom of Bahrain Mo.H. Health Care in the Kingdom of Bahrain. 2012. https://web.archive.org/web/20120227003619/ http://www.gepa2.de/files/Bahrain-Health-Care1.pdf

[28] Alsayed B, et al. Male occult triple-negative breast cancer. BMJ Case Rep. 2019;12(4). 10.1136/bcr-2019-229482 PMC6510126 31005873

[29] Al-Shamsi HO, Abdelwahed N, Al-Awadhi A, Albashir M, Abyad AM, Rafii S, et al. Breast cancer in the United Arab Emirates. JCO Glob Oncol. 2023;9:e2200247. doi: 10.1200/GO.22.00247 36608306 PMC10166434

[30] Xu C, Ganesan K, Liu X, Ye Q, Cheung Y, Liu D, et al. Prognostic value of negative emotions on the incidence of breast cancer: a systematic review and meta-analysis of 129,621 patients with breast cancer. Cancers (Basel). 2022;14(3):475. doi: 10.3390/cancers14030475 35158744 PMC8833353

[31] Bai R, et al. Trends in depression incidence in China, 1990–2019. J Affective Disorders. 2022;296:291–7. 10.1016/j.jad.2021.09.084 34606800

[32] Arabsalmani M, Mohammadian-Hafshejani A, Ghoncheh M, Hadadian F, Towhidi F, Vafaee K, et al. Incidence and mortality of kidney cancers, and human development index in Asia; a matter of concern. J Nephropathol. 2017;6(1):30–42. doi: 10.15171/jnp.2017.06 28042551 PMC5106880

[33] Xie M, Liu X, Cao X, Guo M, Li X. Trends in prevalence and incidence of chronic respiratory diseases from 1990 to 2017. Respir Res. 2020;21(1):49. doi: 10.1186/s12931-020-1291-8 32046720 PMC7014719

[34] Key TJ, Verkasalo PK, Banks E. Epidemiology of breast cancer. Lancet Oncol. 2001;2(3):133–40. doi: 10.1016/S1470-2045(00)00254-0 11902563

[35] Gherasim A, Arhire LI, Niță O, Popa AD, Graur M, Mihalache L. The relationship between lifestyle components and dietary patterns. Proc Nutr Soc. 2020;79(3):311–23. doi: 10.1017/S0029665120006898 32234085 PMC7663317

[36] Neuhouser ML, Aragaki AK, Prentice RL, Manson JE, Chlebowski R, Carty CL, et al. Overweight, obesity, and postmenopausal invasive breast cancer risk: a secondary analysis of the women’s health initiative randomized clinical trials. JAMA Oncol. 2015;1(5):611–21. doi: 10.1001/jamaoncol.2015.1546 26182172 PMC5070941

[37] Iyengar NM, Gucalp A, Dannenberg AJ, Hudis CA. Obesity and cancer mechanisms: tumor microenvironment and inflammation. J Clin Oncol. 2016;34(35):4270–6. doi: 10.1200/JCO.2016.67.4283 27903155 PMC5562428

[38] Autier P, Boniol M, La Vecchia C, Vatten L, Gavin A, Héry C, et al. Disparities in breast cancer mortality trends between 30 European countries: retrospective trend analysis of WHO mortality database. BMJ. 2010;341:c3620. doi: 10.1136/bmj.c3620 20702548 PMC2920378

[39] Arnold M, Morgan E, Rumgay H, Mafra A, Singh D, Laversanne M, et al. Current and future burden of breast cancer: Global statistics for 2020 and 2040. Breast. 2022;66:15–23. doi: 10.1016/j.breast.2022.08.010 36084384 PMC9465273

[40] Heer E, Harper A, Escandor N, Sung H, McCormack V, Fidler-Benaoudia MM. Global burden and trends in premenopausal and postmenopausal breast cancer: a population-based study. Lancet Glob Health. 2020;8(8):e1027–37. doi: 10.1016/S2214-109X(20)30215-1 32710860

[41] Smolarz B, Nowak AZ, Romanowicz H. Breast cancer-epidemiology, classification, pathogenesis and treatment (Review of Literature). Cancers (Basel). 2022;14(10):2569. doi: 10.3390/cancers14102569 35626173 PMC9139759

[42] Jemal A, et al. Global cancer statistics. CA: A Cancer J Clinicians. 2011;61(2):69–90. 10.3322/caac.20107 21296855

[43] Harford JB. Breast-cancer early detection in low-income and middle-income countries: do what you can versus one size fits all. Lancet Oncol. 2011;12(3):306–12. 21376292 10.1016/S1470-2045(10)70273-4

[44] Anderson BO, Cazap E, El Saghir NS, Yip C-H, Khaled HM, Otero IV, et al. Optimisation of breast cancer management in low-resource and middle-resource countries: executive summary of the Breast Health Global Initiative consensus, 2010. Lancet Oncol. 2011;12(4):387–98. doi: 10.1016/S1470-2045(11)70031-6 21463833

[45] Calle EE, Kaaks R. Overweight, obesity and cancer: epidemiological evidence and proposed mechanisms. Nature Reviews Cancer. 2004;4(8):579–91. 15286738 10.1038/nrc1408

[46] Khandekar MJ, Cohen P, Spiegelman BM. Molecular mechanisms of cancer development in obesity. Nat Rev Cancer. 2011;11(12):886–95. doi: 10.1038/nrc3174 22113164

[47] Park J, Morley TS, Kim M, Clegg DJ, Scherer PE. Obesity and cancer--mechanisms underlying tumour progression and recurrence. Nat Rev Endocrinol. 2014;10(8):455–65. doi: 10.1038/nrendo.2014.94 24935119 PMC4374431

[48] Rezakhani L, Darbandi M, Khorrami Z, Rahmati S, Shadmani FK. Mortality and disability-adjusted life years for smoking-attributed cancers from 1990 to 2019 in the north Africa and middle east countries: a systematic analysis for the global burden of disease study 2019. BMC Cancer. 2023;23(1):80. doi: 10.1186/s12885-023-10563-5 36694168 PMC9875390

[49] Johnson KC, Miller AB, Collishaw NE, Palmer JR, Hammond SK, Salmon AG, et al. Active smoking and secondhand smoke increase breast cancer risk: the report of the canadian expert panel on tobacco smoke and breast cancer risk (2009). Tob Control. 2011;20(1):e2. doi: 10.1136/tc.2010.035931 21148114

[50] Gaudet MM, Gapstur SM, Sun J, Diver WR, Hannan LM, Thun MJ. Active smoking and breast cancer risk: original cohort data and meta-analysis. J Natl Cancer Inst. 2013;105(8):515–25. doi: 10.1093/jnci/djt023 23449445

[51] Łukasiewicz S, et al. Breast cancer-epidemiology, risk factors, classification, prognostic markers, and current treatment strategies-an updated review. Cancers. 2021;13(17). 10.3390/cancers13174287 PMC8428369 34503097

[52] Anderson BO, Ilbawi AM, Fidarova E, Weiderpass E, Stevens L, Abdel-Wahab M, et al. The global breast cancer initiative: a strategic collaboration to strengthen health care for non-communicable diseases. Lancet Oncol. 2021;22(5):578–81. doi: 10.1016/S1470-2045(21)00071-1 33691141

[53] Soerjomataram I, Bray F. Planning for tomorrow: global cancer incidence and the role of prevention 2020-2070. Nat Rev Clin Oncol. 2021;18(10):663–72. doi: 10.1038/s41571-021-00514-z 34079102

[54] Clonan A, Roberts KE, Holdsworth M. Socioeconomic and demographic drivers of red and processed meat consumption: implications for health and environmental sustainability. Proc Nutr Soc. 2016;75(3):367–73. doi: 10.1017/S0029665116000100 27021468 PMC4974628

[55] Mizota Y, Yamamoto S. Prevalence of breast cancer risk factors in Japan. J Clin Oncol. 2012;42(11):1008–12. doi: 10.1093/jjco/hys144 22988038

