# Supplementary material for: Burden, and trends of breast cancer along with attributable risk factors in Gulf Cooperation Council countries from 1990 to 2019 and its projections
Source: PLoS One. 2025 Oct 6;20(10):e0331198. doi: 10.1371/journal.pone.0331198 (PMC12500115; doi:10.1371/journal.pone.0331198)
Supplement: S1 Table — (DOCX) [file pone.0331198.s001.docx]

| **S1 Table. temporal trends of ASIR, ASDR, Prevlance, and DALY’s by sex** | | | | | | | | |
| --- | --- | --- | --- | --- | --- | --- | --- | --- |
| **United Arab Emirates** | | | | | | | | |
| **Year** | **ASIR** | | **ASDR** | | **Prevlance** | | **DALY’s** | |
|  | **Female** | **Male** | **Female** | **Male** | **Female** | **Male** | **Female** | **Male** |
| **1990** | 743.75 | 21.86 | 25.49 | 0.96 | 40.77 | 1.21 | 359.00 | 7.81 |
| **1991** | 769.32 | 22.25 | 26.46 | 0.98 | 43.05 | 1.25 | 373.20 | 8.07 |
| **1992** | 794.57 | 22.45 | 27.45 | 0.99 | 45.04 | 1.27 | 386.91 | 8.30 |
| **1993** | 820.01 | 22.68 | 28.47 | 1.00 | 46.86 | 1.29 | 400.08 | 8.52 |
| **1994** | 855.15 | 23.25 | 30.27 | 1.03 | 49.75 | 1.33 | 413.48 | 8.74 |
| **1995** | 877.76 | 23.42 | 31.18 | 1.04 | 51.94 | 1.36 | 425.65 | 8.92 |
| **1996** | 899.31 | 23.63 | 32.04 | 1.05 | 54.01 | 1.39 | 437.44 | 9.09 |
| **1997** | 912.33 | 23.68 | 32.65 | 1.06 | 55.38 | 1.40 | 448.88 | 9.28 |
| **1998** | 920.62 | 23.50 | 33.02 | 1.05 | 56.31 | 1.40 | 460.08 | 9.45 |
| **1999** | 927.23 | 23.52 | 33.33 | 1.05 | 57.35 | 1.41 | 471.09 | 9.60 |
| **2000** | 926.41 | 24.02 | 33.28 | 1.08 | 58.33 | 1.46 | 481.37 | 9.72 |
| **2001** | 922.69 | 23.40 | 33.13 | 1.04 | 59.29 | 1.44 | 494.07 | 9.72 |
| **2002** | 916.37 | 22.92 | 32.87 | 1.02 | 59.58 | 1.42 | 509.40 | 9.69 |
| **2003** | 963.09 | 22.11 | 35.39 | 0.98 | 63.52 | 1.37 | 528.25 | 9.62 |
| **2004** | 1009.94 | 21.66 | 38.28 | 0.96 | 67.80 | 1.34 | 545.85 | 9.56 |
| **2005** | 1014.82 | 21.33 | 38.84 | 0.94 | 69.24 | 1.33 | 556.94 | 9.49 |
| **2006** | 1021.48 | 20.62 | 39.41 | 0.90 | 70.37 | 1.29 | 566.06 | 9.34 |
| **2007** | 1052.00 | 18.99 | 41.13 | 0.81 | 73.07 | 1.18 | 576.76 | 9.05 |
| **2008** | 1047.15 | 18.31 | 41.10 | 0.78 | 72.35 | 1.14 | 584.06 | 8.78 |
| **2009** | 1055.33 | 18.01 | 41.50 | 0.76 | 72.69 | 1.12 | 590.17 | 8.57 |
| **2010** | 1053.72 | 17.87 | 41.31 | 0.76 | 72.36 | 1.11 | 592.19 | 8.48 |
| **2011** | 1053.58 | 17.72 | 41.23 | 0.75 | 73.27 | 1.11 | 585.57 | 8.49 |
| **2012** | 1048.35 | 17.54 | 40.96 | 0.74 | 73.81 | 1.10 | 568.73 | 8.50 |
| **2013** | 1004.19 | 17.54 | 38.27 | 0.73 | 70.85 | 1.11 | 545.97 | 8.51 |
| **2014** | 813.91 | 17.50 | 27.14 | 0.73 | 55.58 | 1.11 | 517.93 | 8.52 |
| **2015** | 808.69 | 17.46 | 26.92 | 0.73 | 55.81 | 1.11 | 510.84 | 8.52 |
| **2016** | 804.62 | 17.33 | 26.75 | 0.72 | 56.13 | 1.11 | 507.55 | 8.41 |
| **2017** | 798.46 | 17.19 | 26.51 | 0.71 | 56.19 | 1.11 | 505.86 | 8.33 |
| **2018** | 795.01 | 17.15 | 26.36 | 0.71 | 56.90 | 1.12 | 511.44 | 8.42 |
| **2019** | 790.98 | 17.12 | 26.19 | 0.70 | 57.48 | 1.13 | 519.28 | 8.55 |
| **Qatar** | | | | | | | | |
| **1990** | 797.44 | 13.01 | 28.21 | 0.76 | 48.92 | 0.76 | 420.40 | 5.52 |
| **1991** | 815.20 | 13.49 | 28.72 | 0.81 | 51.00 | 0.81 | 431.96 | 5.43 |
| **1992** | 834.35 | 13.59 | 29.68 | 0.82 | 52.74 | 0.82 | 442.61 | 5.37 |
| **1993** | 853.51 | 13.30 | 31.12 | 0.79 | 54.22 | 0.79 | 452.35 | 5.32 |
| **1994** | 849.28 | 11.80 | 30.38 | 0.69 | 53.92 | 0.69 | 459.50 | 5.22 |
| **1995** | 847.21 | 11.20 | 29.51 | 0.65 | 54.28 | 0.65 | 465.87 | 5.20 |
| **1996** | 869.97 | 12.68 | 31.18 | 0.77 | 57.63 | 0.77 | 476.34 | 5.39 |
| **1997** | 878.70 | 13.50 | 32.15 | 0.85 | 59.99 | 0.85 | 489.99 | 5.66 |
| **1998** | 875.18 | 13.56 | 32.46 | 0.87 | 61.65 | 0.87 | 506.15 | 5.94 |
| **1999** | 870.79 | 13.10 | 32.80 | 0.85 | 63.11 | 0.85 | 524.46 | 6.19 |
| **2000** | 868.20 | 12.35 | 32.77 | 0.81 | 64.36 | 0.81 | 543.72 | 6.37 |
| **2001** | 880.46 | 11.80 | 33.31 | 0.79 | 67.52 | 0.79 | 569.66 | 6.56 |
| **2002** | 904.54 | 11.83 | 34.70 | 0.82 | 72.62 | 0.82 | 604.50 | 6.80 |
| **2003** | 911.28 | 11.75 | 35.30 | 0.85 | 76.94 | 0.85 | 642.13 | 7.04 |
| **2004** | 910.67 | 11.32 | 35.47 | 0.85 | 80.46 | 0.85 | 678.33 | 7.25 |
| **2005** | 924.36 | 11.80 | 36.24 | 0.91 | 84.59 | 0.91 | 709.77 | 7.46 |
| **2006** | 985.22 | 7.89 | 39.34 | 0.62 | 93.05 | 0.62 | 744.92 | 7.41 |
| **2007** | 1037.12 | 6.94 | 42.07 | 0.56 | 100.92 | 0.56 | 785.49 | 7.58 |
| **2008** | 1043.61 | 6.95 | 42.84 | 0.58 | 104.05 | 0.58 | 823.15 | 7.81 |
| **2009** | 1032.78 | 8.69 | 43.12 | 0.76 | 105.43 | 0.76 | 854.36 | 8.09 |
| **2010** | 1015.13 | 11.72 | 42.91 | 1.08 | 105.75 | 1.08 | 873.42 | 8.39 |
| **2011** | 1005.70 | 9.98 | 42.35 | 0.93 | 107.17 | 0.93 | 883.77 | 8.09 |
| **2012** | 992.54 | 9.13 | 41.57 | 0.87 | 108.36 | 0.87 | 891.40 | 7.71 |
| **2013** | 982.36 | 8.60 | 41.41 | 0.83 | 109.25 | 0.83 | 896.82 | 7.29 |
| **2014** | 971.41 | 8.30 | 41.24 | 0.82 | 109.93 | 0.82 | 900.45 | 6.95 |
| **2015** | 927.80 | 8.13 | 40.04 | 0.81 | 107.27 | 0.81 | 901.39 | 6.81 |
| **2016** | 911.68 | 8.00 | 39.59 | 0.81 | 107.01 | 0.81 | 897.53 | 6.76 |
| **2017** | 892.82 | 7.99 | 38.85 | 0.82 | 106.04 | 0.82 | 891.14 | 6.77 |
| **2018** | 877.01 | 8.17 | 37.91 | 0.84 | 105.18 | 0.84 | 886.59 | 6.92 |
| **2019** | 856.38 | 8.35 | 36.91 | 0.86 | 103.72 | 0.86 | 880.32 | 7.17 |
| **Oman** | | | | | | | | |
| **1990** | 326.04 | 36.92 | 10.94 | 1.84 | 19.29 | 2.31 | 193.95 | 13.60 |
| **1991** | 331.35 | 37.94 | 11.17 | 1.90 | 20.26 | 2.42 | 199.78 | 14.68 |
| **1992** | 342.56 | 38.91 | 11.54 | 1.94 | 21.46 | 2.53 | 206.54 | 15.68 |
| **1993** | 353.01 | 39.77 | 11.90 | 1.99 | 22.63 | 2.62 | 214.27 | 16.57 |
| **1994** | 368.89 | 40.94 | 12.39 | 2.04 | 24.34 | 2.75 | 223.53 | 17.33 |
| **1995** | 383.05 | 42.61 | 12.84 | 2.13 | 26.25 | 2.94 | 234.34 | 17.95 |
| **1996** | 392.08 | 41.84 | 13.17 | 2.09 | 27.75 | 2.95 | 246.23 | 18.34 |
| **1997** | 401.94 | 41.37 | 13.52 | 2.07 | 29.00 | 2.96 | 258.79 | 18.71 |
| **1998** | 413.19 | 40.97 | 13.92 | 2.05 | 30.18 | 2.96 | 271.76 | 19.04 |
| **1999** | 426.28 | 41.04 | 14.37 | 2.06 | 31.86 | 3.01 | 285.12 | 19.36 |
| **2000** | 438.08 | 39.89 | 14.80 | 2.01 | 33.81 | 3.00 | 298.46 | 19.55 |
| **2001** | 445.90 | 38.01 | 15.04 | 1.91 | 35.40 | 2.92 | 311.95 | 19.47 |
| **2002** | 456.50 | 37.12 | 15.44 | 1.87 | 37.05 | 2.89 | 326.19 | 19.24 |
| **2003** | 465.71 | 36.01 | 15.75 | 1.82 | 38.40 | 2.84 | 340.18 | 18.92 |
| **2004** | 470.73 | 34.46 | 15.84 | 1.76 | 39.36 | 2.76 | 353.09 | 18.62 |
| **2005** | 483.36 | 32.48 | 16.35 | 1.65 | 41.12 | 2.62 | 364.97 | 18.42 |
| **2006** | 498.33 | 30.20 | 16.97 | 1.53 | 43.04 | 2.48 | 377.32 | 18.43 |
| **2007** | 515.10 | 30.77 | 17.47 | 1.57 | 44.70 | 2.56 | 390.93 | 18.73 |
| **2008** | 530.96 | 32.25 | 17.99 | 1.64 | 45.99 | 2.69 | 403.90 | 19.10 |
| **2009** | 545.92 | 32.06 | 18.59 | 1.62 | 47.46 | 2.69 | 414.74 | 19.33 |
| **2010** | 535.04 | 32.34 | 18.55 | 1.62 | 47.11 | 2.72 | 420.43 | 19.48 |
| **2011** | 523.34 | 31.58 | 18.31 | 1.60 | 47.14 | 2.70 | 420.99 | 19.49 |
| **2012** | 513.18 | 30.17 | 17.87 | 1.54 | 47.44 | 2.64 | 417.93 | 19.38 |
| **2013** | 482.40 | 29.92 | 17.16 | 1.55 | 45.48 | 2.67 | 412.40 | 19.35 |
| **2014** | 466.51 | 29.40 | 17.01 | 1.55 | 44.64 | 2.67 | 407.96 | 19.31 |
| **2015** | 457.65 | 29.81 | 16.85 | 1.62 | 44.41 | 2.77 | 406.11 | 19.40 |
| **2016** | 448.39 | 29.87 | 16.63 | 1.67 | 44.23 | 2.84 | 406.79 | 19.34 |
| **2017** | 443.88 | 29.27 | 16.47 | 1.63 | 44.39 | 2.81 | 407.91 | 19.18 |
| **2018** | 438.00 | 27.14 | 16.10 | 1.48 | 44.41 | 2.61 | 409.83 | 18.72 |
| **2019** | 434.83 | 26.27 | 15.88 | 1.42 | 44.66 | 2.55 | 413.76 | 18.05 |
| **Kuwait** | | | | | | | | |
| **1990** | 526.53 | 6.79 | 17.74 | 0.50 | 41.35 | 0.50 | 376.74 | 3.78 |
| **1991** | 478.30 | 4.55 | 16.04 | 0.33 | 37.62 | 0.33 | 382.47 | 3.42 |
| **1992** | 450.88 | 3.59 | 14.86 | 0.25 | 35.01 | 0.25 | 389.60 | 3.16 |
| **1993** | 453.10 | 3.20 | 14.57 | 0.23 | 35.83 | 0.23 | 398.22 | 2.99 |
| **1994** | 467.71 | 3.42 | 15.01 | 0.25 | 37.78 | 0.25 | 405.74 | 2.91 |
| **1995** | 498.43 | 4.63 | 16.12 | 0.35 | 40.80 | 0.35 | 410.93 | 2.95 |
| **1996** | 497.55 | 3.69 | 16.24 | 0.27 | 41.34 | 0.27 | 417.20 | 2.92 |
| **1997** | 498.09 | 4.88 | 16.38 | 0.37 | 42.44 | 0.37 | 429.88 | 3.07 |
| **1998** | 520.74 | 3.89 | 17.09 | 0.30 | 45.00 | 0.30 | 445.33 | 3.10 |
| **1999** | 535.24 | 5.25 | 17.49 | 0.41 | 46.48 | 0.41 | 457.90 | 3.25 |
| **2000** | 538.71 | 5.01 | 17.65 | 0.40 | 47.14 | 0.40 | 463.86 | 3.28 |
| **2001** | 549.74 | 3.45 | 18.18 | 0.27 | 48.58 | 0.27 | 464.80 | 3.17 |
| **2002** | 546.65 | 3.54 | 18.19 | 0.27 | 48.01 | 0.27 | 462.88 | 3.15 |
| **2003** | 525.95 | 3.88 | 17.61 | 0.30 | 45.91 | 0.30 | 459.44 | 3.15 |
| **2004** | 530.78 | 3.84 | 17.74 | 0.30 | 46.65 | 0.30 | 457.76 | 3.13 |
| **2005** | 518.55 | 4.83 | 17.38 | 0.39 | 46.56 | 0.39 | 456.58 | 3.18 |
| **2006** | 503.61 | 4.96 | 16.99 | 0.41 | 46.43 | 0.41 | 459.12 | 3.19 |
| **2007** | 525.70 | 5.20 | 18.03 | 0.44 | 49.93 | 0.44 | 467.76 | 3.21 |
| **2008** | 546.09 | 5.25 | 19.17 | 0.45 | 53.33 | 0.45 | 477.59 | 3.23 |
| **2009** | 533.37 | 4.45 | 18.94 | 0.39 | 53.02 | 0.39 | 483.43 | 3.19 |
| **2010** | 503.19 | 4.20 | 17.85 | 0.37 | 50.70 | 0.37 | 483.28 | 3.21 |
| **2011** | 460.07 | 4.29 | 16.41 | 0.39 | 47.20 | 0.39 | 475.00 | 3.30 |
| **2012** | 436.14 | 4.96 | 15.63 | 0.46 | 45.89 | 0.46 | 461.74 | 3.48 |
| **2013** | 407.15 | 5.05 | 14.68 | 0.48 | 44.01 | 0.48 | 445.90 | 3.65 |
| **2014** | 404.24 | 4.63 | 14.69 | 0.45 | 45.18 | 0.45 | 433.36 | 3.78 |
| **2015** | 370.55 | 4.79 | 13.42 | 0.47 | 42.27 | 0.47 | 424.15 | 3.91 |
| **2016** | 361.48 | 4.87 | 13.08 | 0.48 | 41.83 | 0.48 | 419.07 | 4.01 |
| **2017** | 358.19 | 4.92 | 12.95 | 0.49 | 41.90 | 0.49 | 416.49 | 4.09 |
| **2018** | 358.08 | 4.95 | 12.93 | 0.50 | 42.28 | 0.50 | 419.01 | 4.14 |
| **2019** | 358.84 | 5.01 | 13.01 | 0.51 | 42.77 | 0.51 | 423.83 | 4.19 |
| **KSA** | | | | | | | | |
| **1990** | 336.40 | 6.28 | 10.68 | 0.28 | 14.86 | 0.32 | 154.14 | 2.44 |
| **1991** | 354.38 | 6.47 | 11.28 | 0.28 | 16.14 | 0.34 | 156.94 | 2.55 |
| **1992** | 369.59 | 6.61 | 11.80 | 0.29 | 16.97 | 0.35 | 159.72 | 2.65 |
| **1993** | 385.08 | 6.77 | 12.34 | 0.30 | 17.62 | 0.36 | 162.94 | 2.73 |
| **1994** | 403.31 | 7.03 | 12.97 | 0.31 | 18.61 | 0.37 | 167.19 | 2.79 |
| **1995** | 418.37 | 7.21 | 13.50 | 0.32 | 19.78 | 0.39 | 172.67 | 2.84 |
| **1996** | 432.27 | 7.40 | 13.98 | 0.32 | 21.06 | 0.41 | 178.78 | 2.88 |
| **1997** | 442.75 | 7.32 | 14.36 | 0.32 | 21.88 | 0.41 | 184.48 | 2.90 |
| **1998** | 448.54 | 7.14 | 14.59 | 0.31 | 22.26 | 0.41 | 189.94 | 2.90 |
| **1999** | 453.35 | 6.90 | 14.78 | 0.31 | 22.79 | 0.40 | 195.62 | 2.90 |
| **2000** | 451.81 | 6.50 | 14.77 | 0.29 | 23.80 | 0.39 | 201.87 | 2.88 |
| **2001** | 446.21 | 6.04 | 14.63 | 0.27 | 24.64 | 0.38 | 208.59 | 2.84 |
| **2002** | 438.91 | 5.68 | 14.41 | 0.26 | 25.93 | 0.37 | 216.35 | 2.75 |
| **2003** | 431.47 | 5.39 | 14.17 | 0.25 | 26.55 | 0.35 | 224.35 | 2.66 |
| **2004** | 421.52 | 5.12 | 13.85 | 0.24 | 26.97 | 0.34 | 232.83 | 2.58 |
| **2005** | 425.02 | 4.92 | 13.86 | 0.23 | 28.13 | 0.33 | 242.25 | 2.55 |
| **2006** | 428.33 | 4.77 | 13.94 | 0.22 | 29.54 | 0.32 | 253.33 | 2.57 |
| **2007** | 435.29 | 4.66 | 14.18 | 0.22 | 31.07 | 0.32 | 266.36 | 2.62 |
| **2008** | 440.59 | 4.61 | 14.38 | 0.22 | 32.35 | 0.33 | 280.11 | 2.69 |
| **2009** | 440.40 | 4.63 | 14.40 | 0.22 | 33.19 | 0.34 | 293.41 | 2.76 |
| **2010** | 443.74 | 4.86 | 14.53 | 0.23 | 34.56 | 0.36 | 305.75 | 2.83 |
| **2011** | 439.34 | 4.49 | 14.38 | 0.21 | 35.54 | 0.34 | 316.73 | 2.84 |
| **2012** | 437.83 | 4.34 | 14.32 | 0.20 | 36.62 | 0.34 | 327.45 | 2.86 |
| **2013** | 439.79 | 4.36 | 14.36 | 0.20 | 37.74 | 0.35 | 337.93 | 2.89 |
| **2014** | 443.14 | 4.39 | 14.44 | 0.21 | 38.93 | 0.36 | 348.32 | 2.93 |
| **2015** | 441.66 | 4.38 | 14.35 | 0.21 | 39.67 | 0.36 | 358.40 | 2.97 |
| **2016** | 444.80 | 4.41 | 14.42 | 0.21 | 40.76 | 0.37 | 370.02 | 3.03 |
| **2017** | 445.49 | 4.41 | 14.40 | 0.21 | 41.56 | 0.37 | 380.09 | 3.09 |
| **2018** | 446.41 | 4.42 | 14.39 | 0.21 | 42.45 | 0.38 | 387.44 | 3.14 |
| **2019** | 446.15 | 4.43 | 14.35 | 0.21 | 43.07 | 0.39 | 394.18 | 3.19 |
| **Bahrain** | | | | | | | | |
| **1990** | 788.57 | 3.38 | 27.38 | 0.15 | 45.76 | 0.19 | 373.44 | 1.80 |
| **1991** | 803.46 | 3.60 | 28.01 | 0.16 | 47.07 | 0.21 | 376.35 | 1.82 |
| **1992** | 811.94 | 3.68 | 28.47 | 0.16 | 48.03 | 0.21 | 380.03 | 1.85 |
| **1993** | 811.22 | 3.68 | 28.45 | 0.16 | 48.25 | 0.21 | 383.66 | 1.87 |
| **1994** | 801.82 | 3.75 | 28.06 | 0.16 | 47.82 | 0.22 | 386.97 | 1.91 |
| **1995** | 793.17 | 3.85 | 27.77 | 0.17 | 47.69 | 0.23 | 390.29 | 1.95 |
| **1996** | 805.40 | 3.88 | 28.21 | 0.17 | 49.59 | 0.23 | 401.82 | 2.01 |
| **1997** | 809.11 | 3.80 | 27.94 | 0.17 | 51.07 | 0.23 | 423.03 | 2.08 |
| **1998** | 832.84 | 4.07 | 28.63 | 0.18 | 54.37 | 0.26 | 449.53 | 2.19 |
| **1999** | 859.91 | 4.59 | 29.93 | 0.20 | 58.04 | 0.29 | 474.13 | 2.30 |
| **2000** | 872.39 | 4.53 | 30.02 | 0.20 | 59.65 | 0.29 | 488.84 | 2.36 |
| **2001** | 857.60 | 3.58 | 28.87 | 0.15 | 59.66 | 0.23 | 498.19 | 2.36 |
| **2002** | 891.29 | 3.79 | 30.97 | 0.16 | 64.05 | 0.25 | 512.87 | 2.42 |
| **2003** | 901.19 | 4.19 | 32.39 | 0.18 | 66.09 | 0.28 | 526.22 | 2.50 |
| **2004** | 899.94 | 4.45 | 32.98 | 0.20 | 67.24 | 0.30 | 536.66 | 2.58 |
| **2005** | 870.07 | 4.95 | 32.23 | 0.22 | 66.21 | 0.34 | 541.25 | 2.68 |
| **2006** | 845.65 | 4.84 | 31.42 | 0.22 | 66.03 | 0.34 | 542.86 | 2.82 |
| **2007** | 809.05 | 5.10 | 30.30 | 0.23 | 65.28 | 0.37 | 543.08 | 3.04 |
| **2008** | 780.50 | 5.07 | 29.43 | 0.24 | 65.31 | 0.39 | 543.22 | 3.27 |
| **2009** | 750.01 | 5.28 | 28.40 | 0.25 | 64.64 | 0.42 | 542.79 | 3.48 |
| **2010** | 720.88 | 5.84 | 27.54 | 0.28 | 63.31 | 0.47 | 542.55 | 3.63 |
| **2011** | 697.34 | 5.46 | 26.76 | 0.26 | 61.75 | 0.44 | 542.98 | 3.63 |
| **2012** | 686.88 | 5.48 | 26.34 | 0.27 | 61.38 | 0.45 | 544.80 | 3.62 |
| **2013** | 675.62 | 5.17 | 25.73 | 0.25 | 61.09 | 0.43 | 547.30 | 3.57 |
| **2014** | 671.17 | 5.32 | 25.27 | 0.25 | 62.12 | 0.45 | 551.40 | 3.56 |
| **2015** | 664.29 | 5.17 | 24.83 | 0.25 | 62.80 | 0.44 | 556.34 | 3.55 |
| **2016** | 668.93 | 5.12 | 25.12 | 0.25 | 64.13 | 0.44 | 564.89 | 3.58 |
| **2017** | 674.76 | 5.10 | 25.44 | 0.24 | 65.67 | 0.44 | 573.92 | 3.61 |
| **2018** | 666.47 | 5.07 | 25.11 | 0.24 | 66.08 | 0.45 | 581.88 | 3.65 |
| **2019** | 668.34 | 5.05 | 25.16 | 0.24 | 67.50 | 0.45 | 591.53 | 3.70 |

| Table. S1 projection of new cases of breast cancer in GCC countries | | | | |
| --- | --- | --- | --- | --- |
| Country | **Female population** | **Male**  **population** | **Predicted ASIR (female)** | **Predicted ASIR (male)** |
| Bahrain | 613,233 | 961,446 | 50 | 6 |
| Kuwait | 1,828,353 | 2,736,217 | 971 | 16 |
| Oman | 2,051,677 | 3,401,699 | 955 | 58 |
| Qatar | 824,855 | 2,030,407 | 723 | 18 |
| Saudi Arabia | 17,496,049 | 22,965,320 | 9441 | 104 |
| United Arab Emirates | 3,191,516 | 6,812,803 | 1605 | 83 |
| Total | 26,005,683 | 38,907,892 | 13745 new cases | 285 new cases |
