# Supplementary material for: Burden, and trends of breast cancer along with attributable risk factors in Gulf Cooperation Council countries from 1990 to 2019 and its projections
Source: PLoS One. 2025 Oct 6;20(10):e0331198. doi: 10.1371/journal.pone.0331198 (PMC12500115; doi:10.1371/journal.pone.0331198)
Supplement: Attachment — Submitted filename: Reviewer Report for PONE D 25 05700R1 (060925).pdf [file pone.0331198.s003.pdf]

Reviewer Report for PONE-D-25-05700R1
06.09.25

1. General Comments
This paper conducts a thorough analysis of trends in breast cancer incidence, prevalence and death rates, for both women and men across 6 GCC countries for the period 1990-2019. Further, the authors make annual projections age-standardized incidence rates for the next decade (2020-2030) for the same countries. This paper is quite ambitious in comprehensiveness, analyzing the last 20 years and 10-year projections across multiple trends in breast cancer. Reporting these results alone is a great contribution to the literature as well as helping individuals, communities and policymakers in these countries understand recent trends as well as projected trends.

With these primary contributions in mind, I offer the following comments. In particular I address aspects of the paper that are underdeveloped, distract or overreach in terms of author claims.

2. Comment on DALYs analysis throughout paper
Early on in the paper, the authors focus on breast cancer incidence, prevalence and death rates. Not until the Methodology section is the measure of the DALY introduced. (In a similar vein, there are multiple references to prevalence estimates early in the paper though very little is reported on prevalence results such as Fig 2.) The inclusion of DALYs (e.g. Table 1 of Results), seems more a distraction from the main contribution of the incidence and death rates, past and projected. If the DALY burden rates remain in the paper, include explicit motivation in the introduction. Further, guidance should be provided to the reader on these results in the discussion section of the paper.

3. Comment on analysis of results for men
While breast cancer affects both men and women, the magnitude of the incidence, death and DALYs rates for women is significantly higher than for men (Table 1). As such, reporting the average effects is not meaningful. If results for men remain in this paper (see next paragraph), Table 1 might better consist of two panels – reporting for women only and then men only, across the GCC countries.

Factors that may be associated with breast cancer diagnoses for men and women may be quite different. Further, the approach that health care providers may use to support screening and health care service and support is likely to be quite different for men and women. All of this to say that it might be worthwhile to further explore these trends for men only in a separate paper.

4. Comment on attributable risk factors
At several points throughout the paper – e.g., introduction (lines 53-57), references to DALYs in methodology and results sections & discussion – the statements regarding attributable risk factors uses language that makes much more of a causal claim than is suggested by the evidence. The authors make

a stronger case if they reported results as associations or correlations with socioeconomic and environmental factors. If there is significant evidence regarding the causal relationship between one or two of the environmental factors and breast cancer diagnosis, I recommend a more extensive discussion of those factors directly, in the same way they outline exclusion of alcohol consumption from their analysis.

5. Comment on ASIR & HDI
Similar to my previous comment, I am not certain what to make of the statements regarding the relationship between ASIR and the Human Development Index (HDI). At a minimum, more explanation is required to help the reader understand your hypothesis about the association of the ASIR and HDI. How should the reader think about the correlation between these two measures, particularly given that the results (starting line 197) show significant positive correlation for some countries and negative for others. The discussion section (starting line 311)
provides speculation and hypothesizing for those countries with the positive association only. Addressing this may be better suited as a question for future research.

6. Comment on organization of the Discussion section
The discussion section is lengthy and could benefit from the addition of subheadings. This will help guide the reader through your wide-ranging and comprehensive analysis – across gender, time, as well as countries.
As an example, one might break down this section as follows:

Discussion - ASIR for women (line228)
Discussion - ASIR men (line 283)
Discussion - ASIR & HDI (line 311)
Discussion - 10yr Projections (line 342)
Policy Recommendations (line 360)
