# Supplementary material for: Burden, and trends of breast cancer along with attributable risk factors in Gulf Cooperation Council countries from 1990 to 2019 and its projections
Source: PLoS One. 2025 Oct 6;20(10):e0331198. doi: 10.1371/journal.pone.0331198 (PMC12500115; doi:10.1371/journal.pone.0331198)
Supplement: Attachment — Submitted filename: Reviewer Report for PONE D 25 05700R2 (080425).pdf [file pone.0331198.s005.pdf]

Reviewer Report for PONE-D-25-05700-R2
Round 2 revision
08.04.25

Thank you for your responses to my comments on the earlier version and for the opportunity to read the revised paper. As I said in my first report, reporting the trends and 10-year projections in breast cancer incidence, prevalence and death rates for both women and men across 6 GCC countries for the period 1990-2019 is an important contribution. This is particularly salient in countries/regions where disease-specific surveillance monitoring is not likely to be robust.

The following are my replies to the authors' response to items 2-6 in my initial review.

2. Comment on DALYs analysis throughout paper
The authors have addressed my comments regarding the DALY results in both the introduction and discussion.

3. Comment on analysis of results for men
I understand the authors point about inclusion of the data on men. I will leave it to the editor to make the decision whether the paper presents data on both men and women or focus only on women.
If the editor agrees to keep the trends for both men and women, I suggest lines 315-320 be removed from the subsection *ASDR trends in Men* since these lines discuss trends for women and are better placed in the *ASIR (and ASDR) Trends in Women* subsection.

4. Comment on attributable risk factors
The authors have addressed my comments regarding attributable risk factors – meaningfully broadening the discussion and use of a less causal tone in reference to the associations. One exception is line 337 which might be better stated "healthcare infrastructure, which may be associated with increased reported incidence."

5. Comment on ASIR & HDI
The authors have clearly stated the positive association between ASIR and HDI. On lines 208-211, the authors also report the results for ASDR, DALYs to HDI. Further, these negative associations are discussed on lines 343-349 with some speculative discussion that raises more questions for me. Are these results similar for other similar-income countries? How should we think about these opposing (ASIR vs. ASDR) correlations with HDI? I appreciate the authors efforts to use HDI data as a measure to capture any number of socio-economic and cultural factors for a country, however I have concerns that it is too broad or noisy to capture the associations the authors are trying to present.

6. Comment on organization of the Discussion section
The addition of subheadings is helpful to guide the reader and I recommend a couple of additional adjustments.

- ASIR Trends in Women should read ASIR and ASDR Trends in Women as the authors spend considerable time discussing ASDR in this section.
- Switch the order of the last two sections such that Strengths and Limitations come before Policy Implications and Recommendations.
- The last sentence in the Strengths and Limitations is actually a good transition as it makes more of a policy recommendation statement.

NEW Minor edits
In response to reviewers' comments, there appears to be some duplication or lack of transition at some points in the paper.

1. Revise /reduce Lines 71-74 repeated nearly verbatim in the methodology section.

2. Remove line 97 and add DALYs to the list of measures on line 96.

3. Line 105 is the first place the HDI is mentioned as it is not described in the introduction. The HDI is dropped into the paragraph with no transition and no motivation.

4. In the Results section, the authors jump around reporting trends from 1990-2010 and then insert point-in-time estimates for 2019. At a minimum, the section *Sex and age distribution in prevalence, incidence, death and DALYs rate in 2019* should be moved after the *Trends in ASIR, ASDR, prevalence, DALYs by sex from 1990-2010*. However, I am not convinced that the 2019 point-in-time estimates, and Figure 1 provide value-added relative to Table 1 and Fig 2.

5. If trends for men are kept in the paper, I recommend a brief explanation as to why there are not BC ASDR risk factors for men or a similar Fig 4 for men.

6. It would be helpful to the reader to add a top line in Table 3 reporting the actual GBD 2019 observed rate to allow the reader to see the trend 2020-2030 from baseline.

7. Remove discussion of diet and metabolic changes in the Discussion section e.g., Lines 331-335 as it is redundant to the more in-depth discussion in the next section *Metabolic and Lifestyle Factors* starting on line 350.

8. Grammar/typos
The following items were found as I read the R2 version of the paper. They may or may not have been present in my initial review. Nonetheless, I note them here in order to improve the paper.

A. “Data” are plural and should use the plural verb. See for example, line 90. “The primary data ~~was~~ were obtained…”

B. When making relative comparisons of changes across countries, rather than “highest increase” or “highest decrease,” please consider replacing highest with “greatest.” This language is present at multiple points throughout the paper. For the first instance, see the results section of the abstract.

C. Line 333, remove double comma.

D. Line 335, end sentence after “linked to breast cancer” as the remaining words are a hanging clause.

E. Line 373, replace “incidents” with “incidence.”

F. Line 415, remove the preposition “a” such that GBD data as population-level.”

G. Plural of DALY should not include an apostrophe and should read DALYs.
