# Supplementary material for: Burden, and trends of breast cancer along with attributable risk factors in Gulf Cooperation Council countries from 1990 to 2019 and its projections
Source: PLoS One. 2025 Oct 6;20(10):e0331198. doi: 10.1371/journal.pone.0331198 (PMC12500115; doi:10.1371/journal.pone.0331198)
Supplement: Attachment — Submitted filename: Response to Reviewers.docx [file pone.0331198.s002.docx]

**Responses to Reviewer’ Comments**

Ref: [PONE-D-25-05700] - [EMID:7f98d57ddf8330ea]

Dear Editor,

Thank you for the opportunity to submit a revised version of our manuscript, titled " Burden, and trends of breast cancer along with attributable risk factors in Gulf Cooperation Council Countries from 1990 to 2019 and its projections" for consideration in PLOS ONE Journal. We have addressed the majority of the reviewers' suggestions, and these revisions are reflected in the track changes version of the manuscript. A clean version has also been submitted. We trust that the revisions enhance the manuscript and meet the journal’s standards. The authors welcome any further constructive feedback you may have.

Below, we present the reviewers' comments in **black**, followed by our responses in **blue**.

- The manuscript has been revised to comply with the PLOS ONE style requirements, following the structure and formatting guidelines outlined in the PLOS ONE formatting samples as explained in the following links:

<https://journals.plos.org/plosone/s/file?id=wjVg/PLOSOne_formatting_sample_main_body.pdf> and [https://journals.plos.org/plosone/s/file?id=ba62/PLOSOne_formatting_sample_title_authors_affiliations.pdf](https://track.editorialmanager.com/CL0/https:%2F%2Fjournals.plos.org%2Fplosone%2Fs%2Ffile%3Fid=ba62%2FPLOSOne_formatting_sample_title_authors_affiliations.pdf/1/010f0195eb51251d-b0e7a766-c4f4-421d-b756-2b78d1a56309-000000/YzK2862fzfDE-JRtt61AoffnJ41id6GGK_cphmT0ZkU=204)

- We would like to clarify that the data used in our study are publicly available, de-identified, and aggregated datasets obtained from the Global Burden of Disease (GBD) 2019 database. As stated in the Human Participants Research Checklist, this study did not involve direct interaction with human subjects and does not contain personally identifiable information. We also provided a detailed explanation of the data source, acquisition, and processing in the Materials and Methods section. Furthermore, we have included the following statement under the “Availability of data and material” section:

"The data that support the findings of this study are available from the Global Burden of Disease study. No restrictions apply to the availability of these data, which were used under Public Use Files (PUF) data. Data are available at <https://ghdx.healthdata.org/gbd-2019>."

As such, all data used in this study are already freely accessible to the public, and we are fully aligned with your open data policy.

Response to Reviewers' comments

Reviewer's Responses to Questions

**Response**: We sincerely thank both reviewers for their positive evaluations and constructive feedback. We are pleased to note that the manuscript was found to be technically sound, with appropriate statistical analyses and clear data availability. We also appreciate the acknowledgment that the manuscript is intelligibly written and meets the standards of written English.

1. **Reviewer #1:** Many thanks for preparation of this manuscript. I have read it fully. I think it is prepared well and in standard format that is defined for GBD-based manuscript.
   However, please, dear authors are recommended to revise bellow points:
   1. In line 216, "while the heist ASDR uptrend increase", Did dear authors mean "highest"?

**Response:** Thank you for pointing this out. Yes, you are correct — this was a typographical error. We intended to write "highest" instead of "heist." We have corrected this in the revised manuscript (page 9, line 263).

2. I think the manuscript is well informative according to GBD database. However, the article suffers novelty. If dear authors can clarify the novel aspects of the article, it is appreciated to highlight it/them in a proper way.

**Response**: We thank the reviewer for this valuable comment and the opportunity to clarify the novel contributions of our study. We have revised the manuscript to better highlight the originality of our work in both the Introduction (Page 3, line 136-140) and Discussion sections (first paragraph).

3. Please, in "Strength and limitations" section, dear authors can described that how policy makers benefit from information presented in this article.
   **Response**: Thank you for your comment. The "Strength and limitations" section was modified accordingly.

   **Reviewer #2:** This study systematically analyzed the epidemiological trends and risk factors of breast cancer in the GCC countries from 1990 to 2019 and predicted them to 2030. The study fills the gap in breast cancer data in the region and has reference value for public health strategy formulation. The data source is reliable and the method is generally reasonable. For the first time, GBD data were integrated to assess the burden and trend of breast cancer in the GCC countries, and the socioeconomic impact was analyzed in combination with HDI to provide a basis for resource allocation.

**Response:** We sincerely thank the reviewer for their positive and encouraging feedback. We appreciate your recognition of the novelty and value of our work in integrating GBD data to analyze the burden and trends of breast cancer in the GCC region.

1. GBD data rely on estimation models, and the coverage and data quality of local cancer registration systems in GCC countries (such as whether there is underreporting) need to be clearly stated, and their potential impact on the results should be discussed.

**Response:** Thank you for your valuable comment. We fully agree that while the Global Burden of Disease (GBD) study provides a comprehensive and standardized framework for estimating disease burden across regions and time, it relies on modeling techniques that are sensitive to the availability and quality of local data sources. In response to your suggestion, we have now added a dedicated paragraph in the strength and the limitation section to enhance transparency around the data limitations and encourage further investments in strengthening cancer surveillance systems in the region (pages 19-20, line 482–492).

2. The article mentions "excluding alcohol because of low consumption in GCC", but there is a lack of literature support (such as citing WHO or regional survey data) and quantitative evidence (such as the proportion of alcohol attributable in GBD).

**Response:** Thank you for this insightful observation. In response, we have revised the manuscript to include additional references supporting the exclusion of alcohol as a major risk factor in the context of breast cancer in the GCC region.

Please refer to the revised sections on pages 4, line 161 (ref. 9-11).

3. The ASDR of Bahrain men increased significantly (39.13%), while it decreased in other countries, and the possible reasons need to be further explored (such as screening differences, changes in diagnostic criteria, or data heterogeneity).

**Response:** Thank you for your comment. We agree. The necessary changes were implemented accordingly.

Please refer to the revised sections on pages 15, line 389-394.

4. The comparison between the increase in ASDR in Omani women and the decrease in Kuwait women needs to be further explained in combination with medical resources (such as the number of radiotherapy equipment), screening prevalence, or treatment differences.

**Response:** Thank you for your comment. We agree. The necessary changes were implemented accordingly.

Please refer to the revised sections on pages 14&15, line 367-378.
